# Temporal progression of gene regulation of peripheral white blood cells explains gender dimorphism of critically ill patients after trauma

**DOI:** 10.1186/s10020-019-0087-0

**Published:** 2019-05-16

**Authors:** Amol Kolte, Rainer König

**Affiliations:** 1 Integrated Research and Treatment Center, Center for Sepsis Control and Care (CSCC), Jena University Hospital, Am Klinikum 1, 07747 Jena, Germany; 2 Network Modeling, Leibniz Institute for Natural Product Research and Infection Biology-Hans Knöll Institute Jena, Beutenbergstrasse 11a, 07745 Jena, Germany

**Keywords:** Transcriptomics, Gender, Sex, Trauma, Clustering, Gene set enrichment analysis, Sepsis, Systemic infection

## Abstract

**Background:**

The immune response of the critically ill after severe trauma is sex-specific and may explain the different progression of the disease. This may be explained by a different gene regulatory program of their peripheral immune cells. We investigated the progression of the transcription profiles of peripheral immune cells of the patients to elucidate their distinct physiological response and clinical course.

**Methods:**

We compared transcription profiles of whole blood of male and female patients from a larger longitudinal study of critically ill patients after trauma. We developed a statistical analysis pipeline that synchronized the time lapse of the profiles based on the temporal severity score of each patient.

**Results:**

This enabled to categorize the temporal progression of the disease into two pre-acute, an acute and two post-acute phases. Comparing gene regulation of male and female patients at each phase, we identified distinctively regulated molecular processes mainly in the immune response, but also in the regulation of metabolism allowing to cluster these discriminative gene sets into sets of highly related cellular processes. Compared to male patients and healthy controls, female patients showed upregulation of gene sets of innate immunity in the early phase, upregulation of wound healing processes during the acute phase and upregulation of adaptive immunity in the late phase indicating early recovery. In turn, during the pre-acute and acute phase, male patients showed less suppression of gene sets coding for enzymes of energy metabolism and anabolism, most prominently the tricarboxylic acid cycle and β-oxidation, and cellular maintenance, such as cell cycle, DNA replication and damage response, and RNA metabolism.

**Conclusions:**

A stronger innate immune response at the very early phase of the disease may support early clearance of the pathogen and its associated molecular patterns. Upregulation of wound healing processes may explain reduced multiple organ failure during the acute phase. Down regulated energy metabolism during the acute phase may make female patients less susceptible to oxidative stress, the upregulated adaptive immune system reflects an earlier recovery and rebuilding of the adaptive immune system that may protect them from secondary infections. Follow up studies need to be performed confirming these observations experimentally.

**Electronic supplementary material:**

The online version of this article (10.1186/s10020-019-0087-0) contains supplementary material, which is available to authorized users.

## Introduction

Despite recent medical advancements, sepsis and multiple organ failure (MOF) continue to be a major cause of morbidity and mortality in patients surviving severe trauma (WHO 2018). Regardless of the type and severity of the injury, gender dimorphism has been observed in several previous studies. In a large study observing 681,730 trauma patients, female patients showed significantly fewer complications and a 21% lower death rate compared to male patients, despite the same average of injury severity scores (ISS) (Haider et al. 2009). A recent meta-analysis involving 19 studies with 140,328 trauma patients reported less mortality, shorter hospitalization, and fewer complications of female patients (Liu et al. 2015). Consistent with these findings, male gender was associated with a higher risk of major infections and MOF following trauma (Offner et al. 1999; Gannon et al. 2004; George et al. 2003). Mostafa et al. (Mostafa et al. 2002), Deitch et al. (Deitch et al. 2007) and Trentzsch et al. (Trentzsch et al. 2014; Trentzsch et al. 2015) observed that premenopausal female patients are better protected from organ failure and sepsis after critical trauma-induced haemorrhage. In particular, Trentzsch et al. analyzed propensity matched-pairs (*n* = 3887) of male and female severe-trauma patients and observed that pre-menopausal females develop significantly less organ failure despite matching injury, i.e. a matched Abbreviated Injury Scale (for head, thorax, abdomen, extremities), age and co-morbidities (Trentzsch et al. 2015). Although male patients are consistently reported to be associated with increased mortality of hospitalized patients, Rappold et al. (Rappold et al. 2002) observed no difference in mortality between male and female trauma patients. However, their study cohort consisted of rather less severe ill patients with an average mortality rate below 3%. In turn, Napolitano et al. (Napolitano et al. 2001) reported higher mortality in female trauma patients, particularly if they developed pneumonia. Also here, the mortality rate was low (less than 5%). Notably, the injury mechanisms differed in their cohort. Female patients were injured mostly from car accidents while male patients from falls, assaults and motorcycle accidents.

It was reported that female patients respond better to supportive treatments. In a study observing a cohort of more than 4000 trauma patients, premenopausal women had lower serum lactate levels and required less blood transfusion despite more severe injuries (Deitch et al. 2007). In another, prospective clinical study, female patients required lower resuscitation volumes, less inotrope and vasopressor support (36% vs 10%) and less intervention based on the Starling curve to maintain oxygen delivery in the heart compared to similarly injured male patients. The authors concluded that female patients responded better to standardized resuscitation than male patients (McKinley et al. 2002).

Besides these clinical differences, male and female individuals differ fundamentally in their immune response and metabolism. Female individuals show a more robust humoral and cell-mediated immune response, making them less prone to certain infections like tuberculosis, hepatitis B, leptospirosis and other infectious diseases (Klein and Flanagan 2016). They get a better protection from an infection when vaccinated, however they are more prone to autoimmune diseases (Klein and Flanagan 2016). Regarding metabolic differences, studies have shown that energy metabolism differs between sexes as healthy female individuals oxidize more lipids than carbohydrates, they utilize less glycogen from skeletal muscle and produce less hepatic glucose (Tarnopolsky and Ruby 2001). Notably, muscle cells of female individuals have a significantly lower capacity for aerobic oxidation and anaerobic glycolysis (Green et al. 1984).

Trauma can lead to immune dysfunction and cause metabolic derangements. Trauma is often followed by sepsis. The hyper-inflammation during sepsis leads to tissue damage that, in turn, evokes the release of pro- and anti-inflammatory cytokines, but can also suppress a variety of cell-mediated immune responses (Xiao et al. 2011; Desai et al. 2011). Traumatic stress is also known to trigger increased hyperglycaemia, fatty acid oxidation, and decreased ATP production resulting from mitochondrial dysfunction (Marik and Raghavan 2004; Singer 2014). In summary, the immune response and metabolism are severely altered after trauma. Hence, studying the implications of gender are crucial to better understand the underlying pathomechanisms.

Transcriptomics analyses have been successfully used for diagnostics identifying novel virulence factors, predicting antibiotic resistance and studying host-pathogen interactions (Lowe et al. 2017; Leonor Fernandes Saraiva et al. 2017). Also, gender dimorphism of the gene regulatory response to trauma has been investigated. Vught et al. (van Vught et al. 2017) compared gene expression profiles of septic and healthy male and septic and healthy female individuals. Blood of the septic patients was drawn at the day of admission. They identified ERK and MAPK signaling, leukocyte extravasation signaling, PDGF signaling, and ephrin receptor signaling being specifically upregulated in male septic patients, compared to male healthy individuals. They did not find these gene sets to be differentially expressed when comparing female septic patients with female controls. However, they could not detect these differences by a direct comparison. To note, this indirect way of finding male and female specific gene sets may overestimate the regulation in sepsis if the genes in the healthy individuals are low expressed (problem of comparing ratios when there are low numbers in the denominator). Comparing transcription profiles of patients ranging from 12 h to 28 days after severe trauma, Lopez et al. (Lopez et al. 2016) identified gender dimorphism in lymphocyte regulation, response to TGF-β stimulus, ubiquitin dependent protein catabolic processes, and protein or macromolecule catabolic processing. Notably, the authors compared the data from all time points together to identify differentially expressed genes between male and female patients. The temporal progression of the disease was not addressed. These two studies reported gender-dimorphism on a transcriptional level. However, they showed very different results which may be due to neglecting the temporal progression of the disease in each patient. Notably, previous studies on healthy individuals reported that expression changes between sexes considering genes of the autosomal chromosomes are small but are distinctly associated with an increased response to cytokines, response to type I interferon and lymphocyte differentiation in females (Jansen et al. 2014).

We compared the genome-wide gene regulation response to blunt force trauma over time of male and female patients tracking their temporal developments in the transcriptomics of peripheral immune cells as the disease progresses through the early, acute and post-acute phase.

## Methods

### Patient characteristics

The investigated data was taken from the retrospective observational study ‘Inflammation and host response to injury’ (IHRI) (ClinicalTrials.gov identifier: NCT00257231). We accessed this data from the supplementary material of the original publication (Desai et al. 2011). The major inclusion criteria were: blunt trauma without isolated head injury, blood transfusion within 12 h of injury, base deficit > = 6 or systolic blood pressure < 90 mmHg within 60 min after arrival at the emergency department. The major exclusion criteria were: traumatic brain injury (defined as Abbreviated Injury Scale (AIS) score for head > 4 or Glasgow Coma Scale (GCS) motor score < 3 within 24 h of injury), pre-existing immunosuppression or organ dysfunction. These patients were admitted within 12 h after injury and monitored for up to 28 hospital days. For each patient, the first blood sample was taken within 12 h after injury and approximately 1, 4, 7, 14, 21, and 28 days after injury. Leukocytes from whole blood were isolated from peripheral blood samples (more details in ref. (Desai et al. 2011)). Genome-wide gene expression was profiled using Affymetrix HU133 Plus 2.0 GeneChip microarrays. The detailed protocols used for obtaining and processing total blood leukocytes and for array hybridization are described elsewhere (Cobb et al. 2005; Feezor et al. 2004). Among the available data of 168 patients, a subset of 132 (85 male, 47 female) was selected based on their age (between 16 to 50 years) and their maximum MOF score (maximum MOF without neuronal component > 1) during their first 28 days in the hospital. These 132 patients were included in our severity synchronization analysis. The upper age limit of 50 years was set to study and compare pre-menopausal female with male patients. Among these 132 patients, for 129 (83 male, 46 female) there was at least one transcriptomic profile available which could be mapped to the analyzed time window of the synchronized profile. Hence these patients were included in the transcriptomic analysis. A detailed patient selection scheme is given in Additional file 1: Figure S1.

### Data retrieval and pre-processing

Microarray normalization and statistical analysis were performed using R/Bioconductor (https://www.r-project.org). The raw data was background corrected, and normalized by Robust Multi-Array Average normalization employing the *affy* package (Gautier et al. 2004). Only probe sets with a detection significance of *P* ≤ 0.05 were selected for further analysis. Probes were annotated using *lumiHumanIDMapping* and *lumiHumanAll.db* (Du et al. 2008), HGNC symbols were received via Illumina nuIDs. Normalized expression values of probes with the same annotated gene were merged by averaging expression values using the function *avereps* of the *limma* package (Ritchie et al. 2015).

### The workflow

**Fig. 1:**
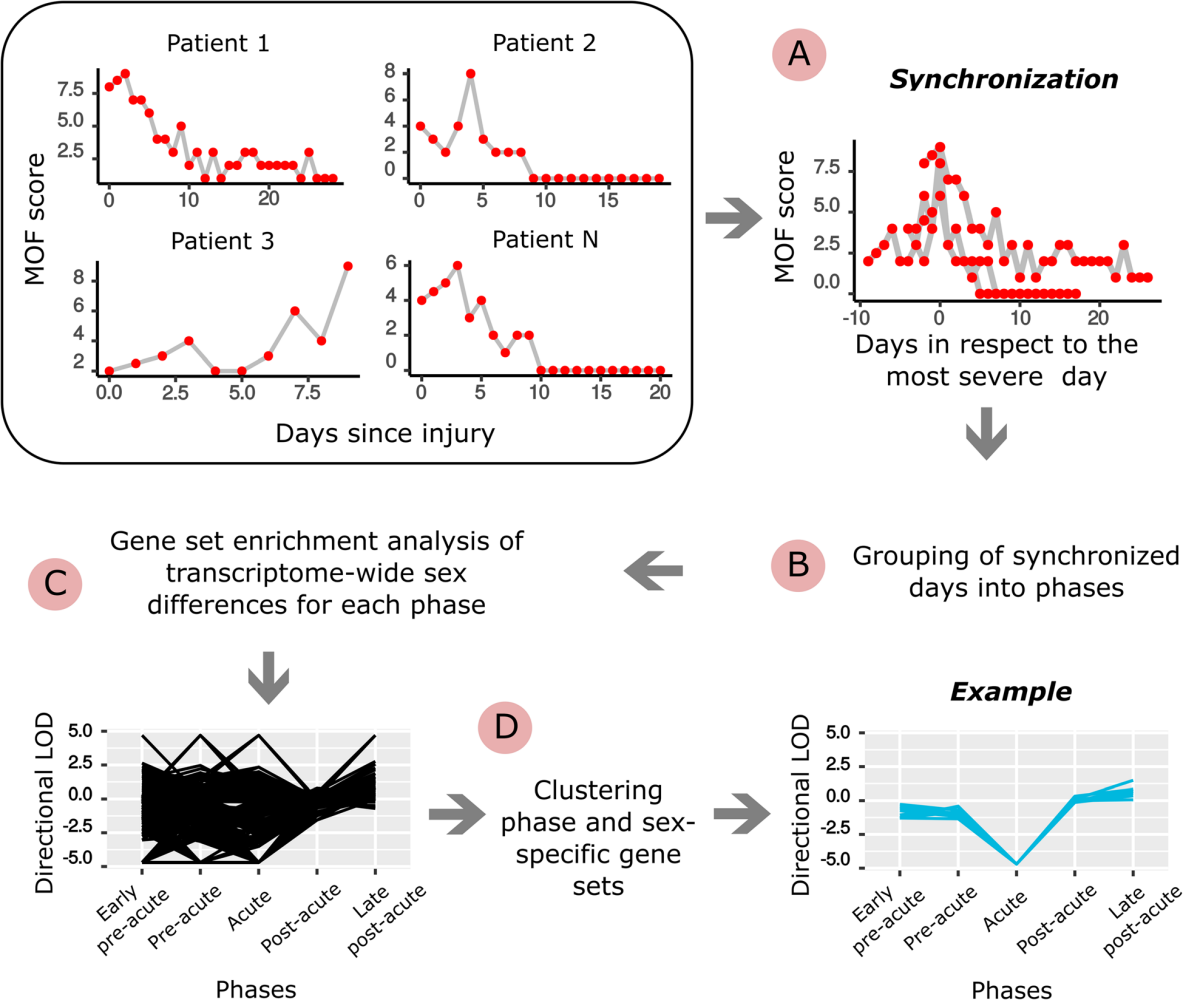
The workflow. **a** Time series expression profiles of the patients were synchronized according to their day of maximal MOF (multiple organ failure) (denoted as acute phase). **b** The investigated days before, at and after the acute phase were grouped into major temporal phases. **c** Gene expression analysis provided sets of differentially expressed genes between male and female patients **d** The identified gene sets were grouped according to their temporal appearance and the similar cellular processes they discribe

The workflow is sketched in Fig. 1 and comprised the following steps:

1. Defining the acute phase: When mapping the expression profiles for each patient over time, we observed that their temporal progression was very heterogeneous. We addressed this issue by synchronizing the profiles taking the severity score of the patients into account. First, for each patient, we identified the time point of his/her most severe state of the disease by selecting the maximal Marshall MOF (Multiple Organ Failure) score (without the neurological component, also in the following) within day 0 to day 28. This selected day, i.e. the day of the highest MOF score was set as the “acute phase” for the according patient. If a patient displayed multiple days the highest MOF score, the time period from the first to the last day with highest MOF score (and maybe days of lower MOF scores in between) was defined as the ‘unstable phase’. An example is given in Additional file 1: Figure S2. Only patients with an unstable phase shorter or equal to 3 days were included in the analysis (*n* = 132) and the whole time from the first to the last day with highest MOF score was regarded as the acute phase. The acute phase was set as a reference for aligning the other days as explained in the next step.

2. Setting the other phases: When regarding the transcription profiles according to this reference day, we observed a strong increase and decrease in severity within 7 days, i.e. from day − 3 to + 3 according to the reference day. Hence, we used this time window for our analysis, i.e. 7 days, 3 days before the acute phase, the day of the acute phase and 3 days after the acute day. For simplification and to get enough samples for each analyzed time point, the window of 7 days was categorized into five major phases. These phases were defined as ‘early pre-acute’ (2 to 3 days prior to the acute phase), ‘pre-acute’ (1 day prior to the acute phase), ‘acute phase’ (the most severe day), ‘post-acute’ (1 day after the acute phase) and ‘late post-acute’ (2 to 3 days after the acute phase). The distribution of the samples in each time point is given in Additional file 1: Table S1, comprising data from altogether 330 samples of 129 patients.

3. Gene expression analysis: Each of the five temporal phases was analysed separately. Differential gene expression between male and female patients was identified by employing Student’s t-test to get the t-values. Only autosomal genes were regarded. The t-values were used for gene set enrichment analysis to select Gene Ontology terms employing the piano package (Väremo et al. 2013) (*n* = 50,000 permutations, selecting ‘mean’ as the gene set statistics, distinct directional, the t-statistic were used from the t-tests). *P*-values resulting from the gene set enrichment tests were corrected for multiple-testing using the method by ‘Benjamini-Hochberg’ (Benjamini and Hochberg 1995).

4. Clustering temporal profiles of enriched gene sets: We clustered gene sets with similar profiles according to their temporal progression. For this, only gene sets were considered which were highly significant (*p* < 0.025) in at least one of the time phases. LOD scores for these gene sets were calculated by LOD = −log_10_(*p),* in which p were the adjusted *p*-values of the considered gene sets. These values were made directional by adding a minus sign for gene sets which were down regulated in female patients. The gene sets were separated into gene sets which were significantly differentially regulated in only one phase (single phase cluster), and gene sets which were significantly differentially regulated in more than one phase (multiple phase cluster). From each of these groups of gene sets, clusters were formed according to the phase of their high significance. These phase-specific clusters were then separated into male and female clusters based on the directional LOD scores. The functional relevence of the clusters was derived based on the biological interpretation of their consisting gene sets.

### Validating our results by regarding transcription profiles from critically ill patients after burn injury

We validated our results with a second publically available dataset. In this study, patients were recruited under the observational and prospective study conducted between 2000 and 2009 in four centres for burn injuries in the U.S. (Seok et al. 2013). These patients were admitted within 96 h after a burn injury of over 20% of the Total Body Surface Area (TBSA). Blood was drawn from the time point of injury until 1 year. The patients underwent at least one excision and grafting surgery. Major exclusion criteria were: associated multiple injuries exclusive of burns (Injury Severity Score (ISS) > =25) and several pre-morbidity conditions (more details are given in ref. (Seok et al. 2013)). In our analysis, we included only patients for which transcription profiles within the first week from the time point of injury were available. A subset of 103 (*n* = 79 male; *n* = 24 female) patients between the age of 16 to 50 years was included in our analysis. The raw data (downloaded from the NCBI GEO, accession number GSE37069) was pre-processed and normalized as described above.

### Additional statistical analyses

All data analyses were performed using the R statistical software environment (https://www.r-project.org/). Distributions were visualized by boxplots of normalized gene expression followed by z-transformation,

1 $$ \mathrm{z}\kern0.5em =\kern0.5em \left(\mathrm{x}\hbox{-} \upmu \right)\kern0.5em /\kern0.5em \upsigma $$

in which x is the normalized gene expression value of gene X, μ is the mean gene expression among all samples of this gene, σ its standard deviation and z the z-transformed value of gene X. Z-transformed expression values are referred to as ‘scaled expression’ values in the following. The trend of MOF scores over time was assessed by a linear regression t-test. Wilcoxon rank-sum test was performed to compare injury severity scores, MOF scores and comparing the distribution of the days of maximal MOF between male and female patients after synchronization. The expression data of the patients was distributed among four different sampling groups. We analysed if this had introduced a batch effect when comparing male with female patients. The batches could have been a serious confounder if samples from male patients had been profiled in other batches as from female patients. Hence, we counted the analysed data of male and female patients in each of the four batches (Additional file 1: Table S2), we found no significant differences (*p* > 0.1 employing a χ^2^ test). To find out if the difference in expression of each of the identified significant gene sets can be explained solely by gender dimorphism, or also by an interaction with the MOF score, we performed an interaction effect analysis employing analysis of variance. A linear model was calculated using the ‘lm’ function, in which the average scaled expression of gene sets was the independent variable, and the MOF score, sex and the ‘product of MOF score and sex’ of each phase were the dependent variables. An F-test was applied to assess the significance of the coefficients. For the analysis of severity-matched patients, a subset of acute phase samples of male and female patients was selected with comparable AIS at baseline and MOF score in their acute phase. The selection was made based on the method of propensity score-matching. A propensity score was calculated for each patient. The patient variables, i.e. AIS at baseline and MOF score in their acute phase were matched for male and female patients employing Matchit (Ho et al. 2011) (method = genetic). Expression data of the balanced subset of male and female samples was analysed to identify significantly enriched gene sets.

## Results

### Synchronizing the temporal progression of the disease and setting the temporal phases

For the studied 132 patients, the difference in their abbreviated injury score (AIS) at admission was non-significant between sexes (male patients: AIS = 3.99 (95% CI, 3.81–4.17); female patients: AIS = 4.15 (95% CI, 3.91–4.39), Additional file 1: Figure S3A). Independent from gender, we observed heterogeneous severity profiles when comparing them in respect to the temporal progression. To address this, we synchronized the profiles matching their day of highest disease severity (peak of the MOF scores, the most severe day) (Fig. 1). After synchronization, the distribution of the day of highest severity was comparable between male and female patients (Additional file 1: Figure S4). Accordingly, also the transcription profiles were synchronized by the day with the maximal MOF score. The synchronization provided a distinct phase of increasing severity (increasing MOF), highest severity (MOF peak) and declining severity (declining MOF), as shown in Additional file 1: Figure S3B.

The patients showed this strong increase and decrease of severity within a 7 days window. As we were most interested in this period, we focussed on this 7-days window, 3 days before the most severe day, the most severe day and 3 days after the most severe day. For 129 patients, 330 transcriptomic profiles were available matching this window. The patient characteristics are given in the Additional file 1: Table S3. During this window, male patients showed a significantly (*p* < 0.01) higher degree of organ dysfunction than female patients. For simplicity, we binned the phases into five sets comprising ‘early pre-acute’ (2 to 3 days prior to the most severe day), ‘acute phase’ (the most severe day), ‘pre-acute’ (1 day prior to the acute phase), ‘post-acute’ (1 day after the acute phase) and ‘late post-acute’ (2 to 3 days after the acute phase).

For each phase, differential expression analysis was carried out to obtain differentially expressed gene sets between male and female patients. These gene sets were grouped according to their significance across the temporal phases. The significant gene sets of all phases are listed in Additional file 1: Table S4. We describe these gene sets according to their chronology and after clustering them into sets of similar biological processes.

### The innate immune response is upregulated in the *early pre-acute phase* of female patients

**Fig. 2:**
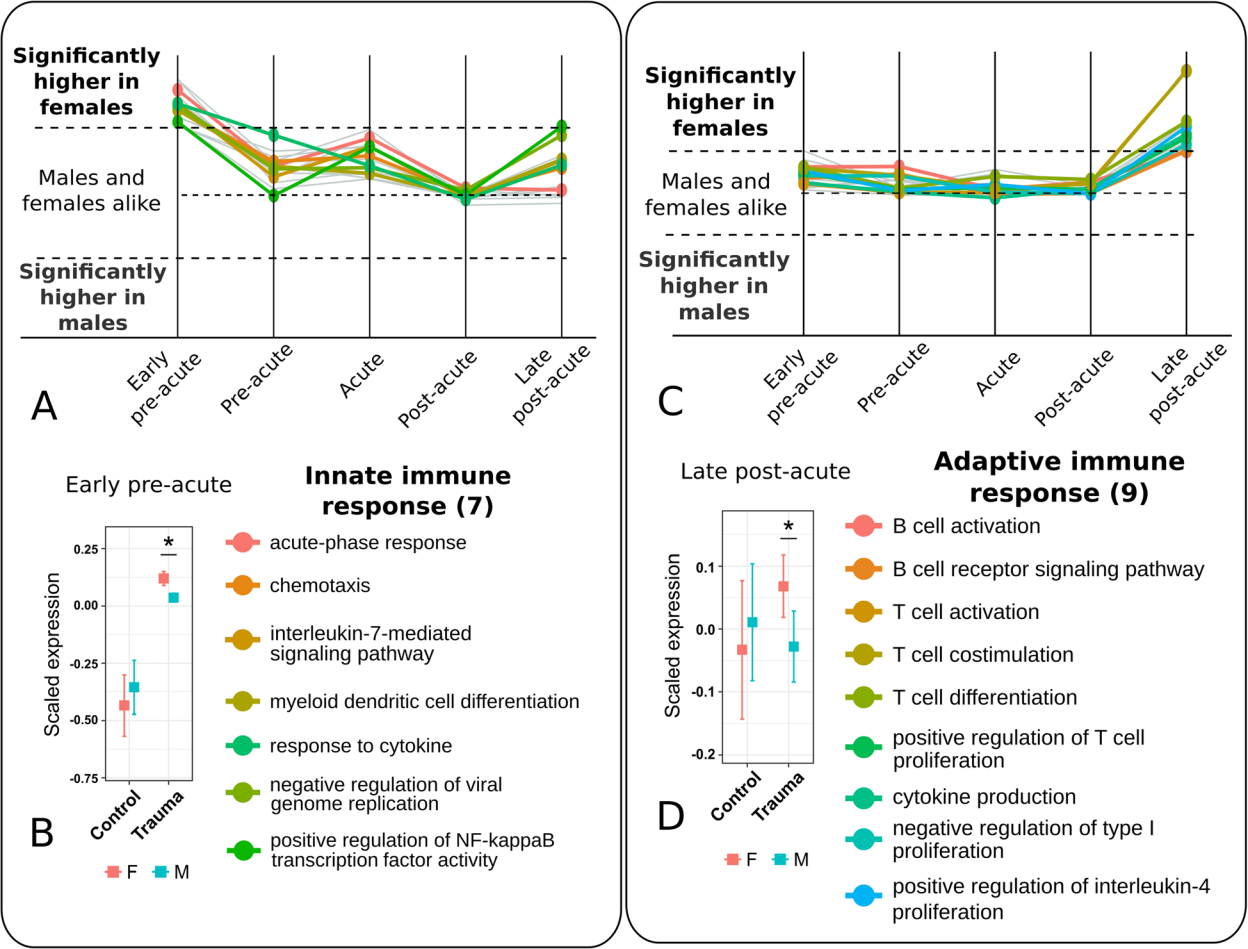
Gene set clusters of the early pre-acute (left) and late post-acute phase (right). **a** The cluster of the early pre-acute phase consisted of 16 gene sets being upregulated in female patients. They comprised seven gene sets of the innate immune response. These gene sets are shown here. The complete list is given in Additional file 1: Fig. S5. **b** For the same phase, in comparison to healthy controls, leukocytes of both sexes showed a higher expression of genes for the innate immune response. A distinctively higher expression was observed in female patients. **c** Gene set clusters of the late post-acute phase consisted of 17 gene sets being upregulated in female patients, nine were related to the adaptive immune response. These gene sets are shown here. The complete list is given in Additional file 1: Fig. S12. **d** For the same phase, in comparison to healthy controls, leukocytes of female patients showed upregulation of the adaptive immune response. These gene sets were similarly expressed in male patients and healthy controls. The z-transformed expression values were referred to as ‘scaled expression’ values. Significance of difference between the expression of genes of male and female individuals was tested using two-sided Student’s t-tests (* *P* < 0.05, mean bar and Whiskers of the boxplots: mean ± se)

The early pre-acute phase was defined as the period of 2 to 3 days prior to the acute phase. As expected from the synchronization, there was a significant (*p* < 0.0001) rate of increase in the MOF score from this phase to the acute phase across male and female patients. The comparison of the transcription profiles at this phase revealed a distinct higher regulation of gene sets associated with an *acute-phase response* in female patients compared to male patients. A total of seven gene sets related to the innate immune response were identified in this cluster specific to females in the early pre-acute phase. These included *positive regulation of NF-κB transcription activity*, *myeloid dendritic cell differentiation* and *chemotaxis, cytokine response*, and, more specifically, *IL-7 mediated signaling*. Figure 2a shows the temporal profiles of all identified gene sets corresponding to innate immune response. Additional file 1: Figure S5 shows all gene sets which were upregulated in females during this phase.

We identified another cluster of gene sets which were upregulated in leukocytes of female patients. Gene sets in this cluster were upregulated in at least two of the first three temporal phases of the disease, i.e. in the early pre-acute, pre-acute and acute phases. Also, this cluster contained immune system related gene sets, such as *cellular response to tumour necrosis factor*, *positive regulation of ERK1-ERK2 cascade*, and *neutrophil chemotaxis*. Additional file 1: Figure S6 shows the temporal profiles of all gene sets in this cluster. We compared the expression of these seven gene sets of innate immunity from the first cluster to their expression in healthy controls and observed that in both sexes these gene sets were upregulated, and in the female patients, these sets were significantly more upregulated (Fig. 2b). The other gene sets of both clusters contained sets for further signaling processes like phosphatidylinositol mediated signaling, transcriptional regulatory processes, protein modification and neural processes (Additional file 1: Figures S5 and S6).

In summary, in the early pre-acute phase, genes coding for the innate immune response were upregulated in female compared to male patients, and particularly, of the innate immune system.

### Wound healing and recovery processes are upregulated early in female patients

**Fig. 3:**
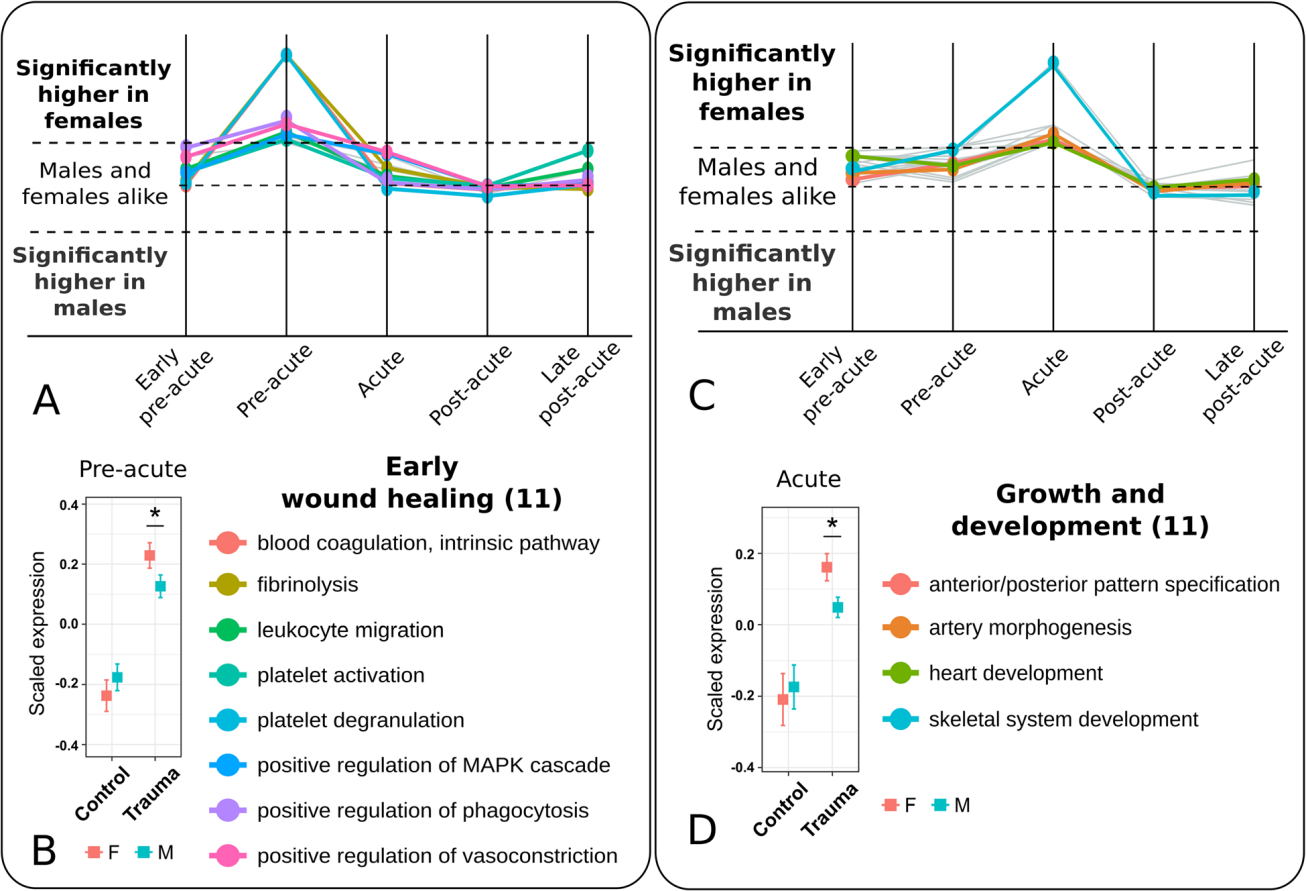
Gene set clusters of the pre-acute (left) and acute phase (right). **a** The cluster of the pre-acute phase contained 11 gene sets being upregulated in leukocytes of female patients. These gene sets are all related to wound healing processes. Eight representative gene sets are shown here. The complete set is given in Additional file 1: Fig. S7. **b** In comparison to healthy controls, the gene sets were higher expressed in the leukocytes of both sexes. A distinctively higher expression was observed in the leukocytes of female patients. **c** The cluster of the acute phase contained 19 gene sets being upregulated in female patients. Eleven of these were associated with growth and development. Four representative gene sets are listed here. The complete list is given in Additional file 1: Fig. S8. **d** In comparison to healthy controls, leukocytes of both sexes showed a higher expression of these gene sets, distinctively higher expression was observed in leukocytes of female patients. The z-transformed expression values were referred to as ‘scaled expression’ values. Significance of difference between the expression of genes of male and female patients was tested using two-sided Student’s t-tests (* *P* < 0.05, mean bar and Whiskers of the boxplots: mean ± se)

During the pre-acute and the acute phase, we observed a distinct upregulation of recovery processes in female patients. The upregulated gene sets at the pre-acute phase were predominantly associated with the stages of early wound healing such as tissue restoration and blood coagulation. These included, *positive regulation of vasoconstriction, platelet activation, blood coagulation, intrinsic pathway* and *platelet degranulation.* The process of *fibrinolysis* was also upregulated in female patients. Simultaneously, we observed an upregulation of cellular processes governing *leukocyte migration* and *positive regulation of phagocytosis,* together with signaling processes such as *positive regulation of MAPK cascade* in female patients. There were eleven gene sets identified in this cluster related to the early wound healing response. Figure 3a shows the temporal profiles and lists of representative gene sets. The complete cluster is given in Additional file 1: Figure S7. During the acute phase, the most prominent upregulated gene sets in leukocytes of female patients were growth and development related gene sets. These included *anterior/posterior pattern specification*, *artery morphogenesis*, *skeletal system development* and gene sets for several neural processes. Upstream to these, *response to IL-1 signaling* and *cytokine-mediated signaling* were also upregulated. This cluster consisted of eleven gene sets for growth and developmental processes. Figure 3c shows the temporal profiles and lists of representative gene sets, while the complete cluster is given in Additional file 1: Figure S8. To note, the eleven gene sets for early wound healing in the pre-acute phase and further eleven gene sets for growth and developmental processes in the acute phase were upregulated in male and female trauma patients, when compared to *healthy* individuals. Still, these gene sets showed a significantly higher expression in female compared to male patients (Fig. 3b, d).

### During the *pre-acute* and *acute phases,* genes for transcription and translation, cell cycle, DNA damage and repair and oxidative phosphorylation are downregulated in female patients

**Fig. 4:**
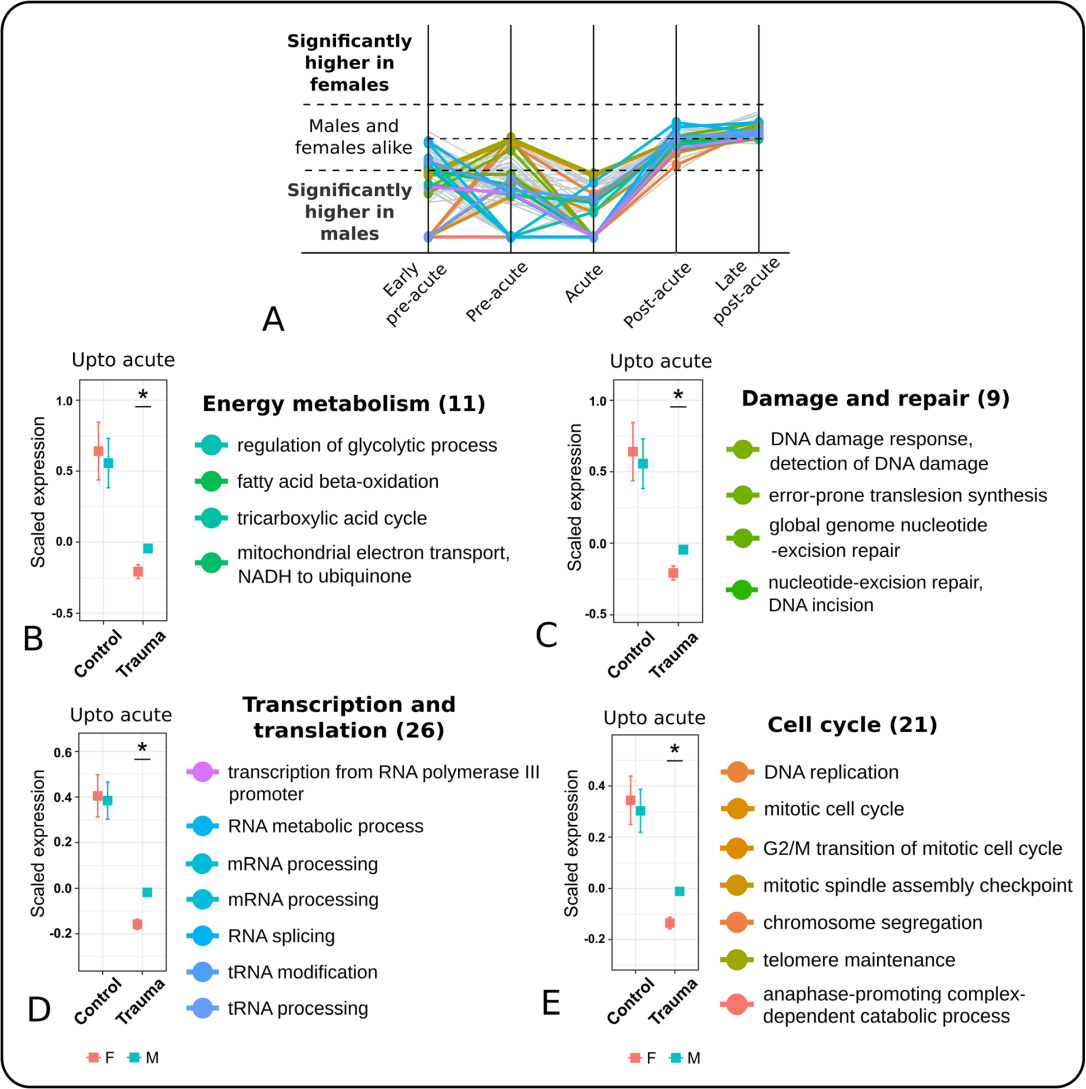
The gene set cluster of the early pre-acute, pre-acute and acute phase. **a** The cluster consisted of 85 gene sets being downregulated in female patients. Most of these were associated to **b** energy metabolism and the mitochondrion (11 gene sets), **c** cell cycle (21 gene sets), **d** transcription and translation (26 gene sets), and **d** damage and repair (9 gene sets). For each category, their representative gene sets are listed here. The complete list is given in Additional file 1: Fig. S10. In comparison to healthy controls, for all categories, leukocytes of both sexes showed a lower expression of these gene sets, but relatively lower expression was observed in female patients. The z-transformed expression values were referred to as ‘scaled expression’ values. Significance of difference between the expression of genes of male and female patients was tested using two-sided Student’s t-tests (* *P* < 0.05, mean bar and Whiskers of the boxplots: mean ± se)

Two third of the identified sex specific transcriptomic gene sets were *downregulated* in female compared to male patients. Most of these gene sets were observed within the first three phases, i.e. during the early pre-acute, pre-acute and acute phases. We grouped these gene sets into two larger clusters. One cluster comprised gene sets that were downregulated in at least two of these phases (Fig. 4), the other cluster comprised gene sets which were downregulated only in one phase, i.e. the acute phase (Additional file 1: Figure S9).

The first cluster contained gene sets related to transcription and translation, cell cycle, DNA damage and repair and energy metabolism including oxidative phosphorylation (OxPhos). Compared to healthy individuals, the expression of these gene sets was downregulated in both sexes of the patients. Figure 4 shows the temporal profiles and their representative gene sets. The complete cluster is given in Additional file 1: Figure S10. Still, these gene sets were distinctively more down regulated in female than in male patients. ATP producing processes such as *regulation of glycolytic processes*, *TCA cycle*, *fatty acid beta-oxidation* and *electron transport chain* were significantly down regulated in female, compared to male patients. In line, ATP consuming processes were also downregulated in female patients, such as DNA repair by excision mechanisms, RNA processing, and specifically *transcription* via *RNA polymerase I, II and III*, *RNA transport*, *RNA splicing*, *tRNA processing*, *tRNA modification*, *RNA metabolism* etc. and gene sets for cell cycle comprising *DNA replication* to *telomerase maintenance*, *transition of mitotic cell cycle*, *mitotic spindle assembly checkpoint*, *chromosome segregation* and *anaphase promoting complex-dependent catabolic process*. The second cluster comprised gene sets which were again related to transcription and translation, DNA damage and repair, and energy metabolism (Additional file 1: Figure S9). In comparison to healthy individuals, these gene sets were downregulated in male and female patients during the acute phase, except for energy metabolism related gene sets, which gene expression in male patients and healthy individuals was comparable (Additional file 1: Figure S11). In summary, cell cycle, repair, and energy producing and ATP consuming processes were distinctively downregulated in female, compared to male patients in the pre-acute and acute phases. This contrasted with the upregulation of these processes after the acute phase (see next section).

### Genes coding for the adaptive immune response are distinctively higher expressed in leukocytes of female patients in the late post-acute phase

After the acute phase, as expected from the synchronization, the MOF scores declined at a significant rate (*p* < 0.0001) reflecting a phase of recovery in male and female patients. For female patients, we observed this also on the molecular level. Particularly in the late post-acute phase, i.e. 2 to 3 days after the acute phase, we observed distinct upregulation of genes of the adaptive immune system in female patients. This comprised *T cell activation*, *T cell co-stimulation*, *T cell differentiation* and *positive regulation of T cell proliferation*. Additionally, in female patients we observed a distinct upregulation of *cytokine production*, and particularly of *IL-4 production*, together with the upregulation of *negative regulation of type I interferon production*. We also observed gene sets for *B cell activation*, *B cell receptor signaling* to be upregulated in female, compared to male patients. Figure 2c shows the temporal profiles of gene sets from the adaptive immunity cluster. This cluster consisted of nine gene sets out of which five representatives are listed. The complete cluster is given in Additional file 1: Figure S12. Comparing the expression profiles to the profiles of healthy individuals, showed that these nine gene sets for adaptive immunity where upregulated only in female patients. Male patients expressed these comparably to the controls (Fig. 2d). In summary, leukocytes of female patients showed a distinct higher expression of genes being responsible for restoring the adaptive immune system during this post-acute phase.

### The divergent regulation can be attributed to gender dimorphism rather than to the different disease severities

Male patients showed more MOF compared to female patients at the acute phase. Thus, the identified transcriptomic differences between male and female patients could have been due to the differences in the severity but not directly associated with gender dimorphism. To justify if the identified transcriptomic differences can be attributed to the sexes but not to the severity alone, an analysis was performed in which we selected a subset of transcription profiles (*n* = 28 female, *n* = 34 male) from the acute phase in such a way that the MOF scores and AIS scores of the corresponding male and female patients were comparable at the acute phase. Comparing the transcriptional profiles of male *versus* female samples showed 123 gene sets to be significantly differentially expressed (Additional file 1: Table S5), out of which 114 (93%) were identical to the previously identified gene sets during the acute phase. As expected, the downregulated gene sets in female patients were associated with transcription and translation, cell cycle, DNA damage and repair and oxidative phosphorylation, while upregulated gene sets were related to innate immune response, and growth and development. To further clarify if the identified gene sets depended on the according MOF scores rather than the sex itself, we performed an interaction analysis between MOF and sex in each phase. This showed a significant dependence on sex and rather independence of the MOF scores. (Additional file 1: Figure S13). In summary, the identified gene sets were not due to the differences in the severities but the gender dimorphism itself.

### Similar gender dimorphism of gene regulation in blood of trauma patients and patients with severe burn injury

We studied the transcription profiles of patients with severe burn injury (taken from a recent publication by Seok et al. (Seok et al. 2013)). The studied patients were 16–50 years old with injury severity scores above 25. As the longitudinal severity scores were not available for synchronization, we studied the transcriptomic response of the first week after burn injury. We observed that the transcriptomic gender dimorphism was comparable to the pre-acute and acute phase of trauma patients. This included upregulation of the innate immune response, wound healing and growth processes, and downregulation of transcription and translation, cell cycle, DNA damage and repair and energy metabolism in female patients with burn injury (Additional file 1: Figure S14). The adaptive immune response in these patients was overall suppressed and identical between sexes during the first week of burn injury. Notably, female patients showed significant upregulation of the adaptive immune response after the first week. These results support the results we observed for patients after blunt trauma.

## Discussion

Previous clinical trials showed that female (in their premenopausal phase) compared to male trauma patients are better protected from organ failure, show fewer complications, better tolerate critical trauma and develop less severe organ failure. As differences in gene regulation of healthy individuals have been described (Klein and Flanagan 2016; Tarnopolsky and Ruby 2001; Green et al. 1984; Wu and O’Sullivan 2011), we hypothesized that gender dimorphism is also prominent in the gene regulation of peripheral leukocytes of the critically ill and may explain the better clinical course of female patients. As a case study, we investigated critically ill patients after blunt trauma. We developed a statistical framework synchronizing the transcription profiles based on the severity of the patients which enabled us to track differences in male and female patients before, at, and after the most acute phase of organ dysfunction. By this, we detected a very divergent regulation of male and female patients. As the findings were derived from trauma patients with significant heterogeneity and limited stratified samples in some temporal phases, we validated the main findings by a second, independent dataset of transcription profiles from peripheral leukocytes of patients with severe burn injuries. Male patients had a higher MOF score compared to female patients. This may have been a confounder in our analysis. Hence, we investigated if the results can be replicated upon comparing male and female patients of comparable highest MOF scores. We selected such a balanced sub-cohort by employing propensity score matching. We got nearly the same significantly distinct gene sets as for the non-balanced cohort, confirming that the divergent regulation can be attributed to gender dimorphism rather than to the different disease severities.

In the early pre-acute phase, leukocytes of female patients showed a stronger innate immune response at the transcriptional level. Here, we observed upregulation of NF-κB transcription activity and the ERK1-ERK2 signaling cascade. The role of NF-κB and ERK1-ERK2 signaling are well described in the survival, activation and differentiation of innate immune cells independent of gender (Buscà et al. 2016; Liu et al. 2017)*.* Distinct gender dimorphism had been described for components of innate immunity such as higher efficiency of antigen presenting cells (APCs), higher activation and phagocytotic activity of macrophages and neutrophils of female patients (Klein and Flanagan 2016). This supports our observations on a systems-view. We observed upregulation in female patients of genes for higher myeloid dendritic cell differentiation and neutrophil chemotaxis. Myeloid dendritic cells are the bridge linking innate and adaptive immunity. They comprise a heterogeneous population of cells presenting antigens to T-cells (Chistiakov et al. 2015). During the same phase, we observed IL-7 mediated signaling to be upregulated in female patients. IL-7 signaling has been extensively studied in the context of survival and differentiation of B-cells, and proliferation of B- and T-cells (Sammicheli et al. 2012; Corfe and Paige 2012; Guimond et al. 2009). Immunotherapy by application of IL-7 has been shown to enhance the immune response in patients with limited naïve T-cells (Unsinger et al. 2010; El-Kassar et al. 2012; Tuckett et al. 2014). Furthermore, high levels of IL-7 have been reported to affect the selection of the T- *versus* the B-cell lineage (El-Kassar et al. 2012). These early triggers may support the recovery of adaptive immunity which we found upregulated in female patients in the *post-acute* phase.

We observed upregulation of early wound healing and recovery processes in female patients during the *pre-acute* phase. Better wound healing in premenopausal females has been evidenced in a few studies before (Jorgensen et al. 2002; Ashcroft and Ashworth 2003; Gilliver et al. 2008). Wound healing is a highly organized process involving several characteristic overlapping steps comprising restoring skin and vessel integrity, inflammation for attracting leukocytes, proliferation to diminish the lesioned tissue area and remodelling of the extra cellular matrix (Gonzalez AC de et al. 2016). Indeed, in the pre-acute phase, we observed that leukocytes of female patients upregulated processes associated with restoring tissue and vessel integrity. These included upregulation of platelet activation/degranulation and blood coagulation to restrict losing blood. The second stage of wound healing is characterized by localized swelling and clearance of damaged cells and pathogens from the wound area. In line, leukocytes of female patients showed an upregulation of genes for leukocyte migration and regulation of phagocytosis. Higher activation and phagocytotic activity of macrophages and neutrophils have been previously reported in females (Klein and Flanagan 2016). We observed upregulation of the *MAPK cascade* and required *protein phosphorylation* gene sets in female patients, which play a predominant role for cell proliferation, cell-cell adhesion and growth during early wound healing (Thuraisingam et al. 2010). It is reasonable that, at the transcriptomic level, healing processes are initiated before the acute phase. These processes may form the functional basis to initiate wound healing processes. Upregulation of these processes was followed by upregulated gene sets related to cell growth and development at the acute phase*.* Leukocytes of female patients showed an upregulation of IL-1 signaling. The family of IL-1 cytokines activates the innate immune system and also supports the activation and proliferation of T-cells (Sims and Smith 2010). In line, we observed upregulation of the adaptive immune system and T-cell growth in the late post-acute phase.

The differential regulation of energy metabolism and housekeeping processes covered almost two-third of the identified sex specific gene sets. From the pre-acute to the acute phase, leukocytes of female patients downregulated energy metabolism and, in particular, oxidative phosphorylation. We observed downregulation of ATP producing *and* consuming processes. Surprisingly, peripheral leukocytes of female patients may sustain a limited metabolism by such downregulation without being driven into apoptosis, as we did not observe upregulation of cell death associated processes compared to male patients.

During the pre-acute phases of increasing multiple organ failure, the reduced energy production may be an advantage by limiting the production of reactive oxygen species (ROS). It was reported that cells of females are less exposed to oxidative stress in healthy conditions. Ide et al. observed a lower abundance of in vivo biomarkers for oxidative stress in premenopausal women (Ide et al. 2002). In an animal model, lower oxidative stress was observed (Barp et al. 2002), and ROS production was lower in endothelial cells of female compared to male individuals (Zhang and Lingappan 2017). Hence, higher exposure to oxidative stress may be an intrinsic risk factor for male individuals when getting critically ill. In his review about the discrepancy between the need for energy and the potential risk of cell damage when producing energy, Mervyn Singer describes that a hallmark of survivors of sepsis is to better preserve ATP and mitochondrial functions (Singer 2014). He suggests that in these patients, cells may enter a “hibernating state in the face of overwhelming inflammation”. Our observation supports this as particularly in the critical pre-acute phases, we observed a distinct downregulation of these energy producing and consuming processes in the leukocytes of female patients. Taken together, the leukocyte transcriptomic response to trauma point to a better bioenergetic tolerance and oxidative damage resistance in female patients.

We observed that gene sets of the adaptive immune response were distinctively higher expressed in leukocytes of female, compared to male patients during the late post-acute phase, and also compared to healthy controls*.* Additionally, female patients showed a distinct upregulation of IL-4 production. The presence of IL-4 during the response of naïve T helper cells has been shown in the development of Th2 cells (Zhu et al. 2010). Interestingly, negative regulation of type I interferon production was also upregulated in female patients. Type I IFNs are known to be important for the host defending viruses. However, in vivo studies have also identified their immune suppressive mechanisms (McNab et al. 2015), hence low levels for IFN type one’s negative regulation may support the immune recovery. This suggests that gene regulation in female, but not in male patients, paves the way for better recovery of the adaptive immune system at the later stages.

There are certain limitations of our study. The patients with multiple acute phases that were more than 3 days apart could not be considered in the study due to their limited number and the availability of their transcriptome data. The primary goal of our study was to identify longitudinal differences of the regulatory response of peripheral leukocytes between male and female trauma patients. A higher number of time-resolved transcription profiles followed up by functional studies in vitro and in animal models may provide a more comprehensive view of the complicated response and the following recovery processes.

## Conclusion

After critical trauma, female and male patients exhibited a distinctively different transcriptomic behaviour. Before the most severe day (i.e. the day with the highest MOF), female patients showed differential regulation for a stronger innate immune response, before, and at the most severe day better bioenergetic tolerance and better oxidative damage resistance. They showed early upregulation of wound healing mechanisms and, after the most severe day, a distinct upregulation of the adaptive immune system. These results support our understanding of the better clinical course of female patients after trauma.

## Additional files

Additional file 1: **Figure S1.** Workflow for selecting the patients. **Figure S2.** Definition and distribution of unstable phases. **Figure S3.** Comparison of the AIS scores at baseline and Marshall MOF severity profiles after synchronization. **Figure S4.** Comparison of the day of highest severity (Marshall MOF) in male and female patients. **Figure S5.** Complete cluster of upregulated gene sets in females in the early pre-acute phase. **Figure S6.** Complete cluster of upregulated gene sets in females in at least two phases among the early pre-acute, pre-acute and acute phase. **Figure S7.** Complete cluster of upregulated gene sets in females in the pre-acute phase. **Figure S8.** Complete cluster of upregulated gene sets in females during the acute phase. **Figure S9.** Complete cluster of downregulated gene sets in females during the acute phase. **Figure S10.** Complete cluster of downregulated gene sets in females in at least two phases among the early pre-acute, pre-acute and acute phase. **Figure S11.** Scaled expression of energy metabolism related genes in the acute phase. **Figure S12.** Complete cluster of upregulated gene sets in females in the late post-acute phase. **Figure S13.** Average scaled expression of the identified sex-specific gene sets in relation to the MOF and sex during the defined phases of the severity. **Figure S14.** Scaled expression of the identified sex-specific gene sets in the early pre-acute, pre-acute, acute and late post-acute phases of patients with burn injury. **Table S1.** Distribution of microarray samples across the phases. **Table S2.** Distribution of microarray samples across the sampling groups. **Table S3.** Patient characteristics. **Table S4.** List of significant gene sets in all investigated phases. **Table S5.** List of significant gene sets in the acute-phase of the propensityscore matched subset of male and female patients. (DOC 4496 kb)

## Abbreviations

AIS: Abbreviated Injury Scale
GCS: Glasgow Coma Scale
HGNC: HUGO Gene Nomenclature Committee
IL: Interleukin
LOD: Log of Odds
MOF: Multiple Organ Failure
ROS: Reactive Oxygen Species
TBSA: Total Body Surface Area
