# Supplementary material for: Temporal progression of gene regulation of peripheral white blood cells explains gender dimorphism of critically ill patients after trauma
Source: Mol Med. 2019 May 16;25:19. doi: 10.1186/s10020-019-0087-0 (PMC6521436; doi:10.1186/s10020-019-0087-0)
Supplement: Supplementary file 1 — Figure S1. Workflow for selecting the patients. Figure S2. Definition and distribution of unstable phases. Figure S3. Comparison of the AIS scores at baseline and Marshall MOF severity profiles after synchronization. Figure S4. Comparison of the day of highest severity (Marshall MOF) in male and female patients. Figure S5. Complete cluster of upregulated gene sets in females in the early pre-acute phase. Figure S6. Complete cluster of upregulated gene sets in females in at least two phases among the early pre-acute, pre-acute and acute phase. Figure S7. Complete cluster of upregulated gene sets in females in the pre-acute phase. Figure S8. Complete cluster of upregulated gene sets in females during the acute phase. Figure S9. Complete cluster of downregulated gene sets in females during the acute phase. Figure S10. Complete cluster of downregulated gene sets in females in at least two phases among the early pre-acute, pre-acute and acute phase. Figure S11. Scaled expression of energy metabolism related genes in the acute phase. Figure S12. Complete cluster of upregulated gene sets in females in the late post-acute phase. Figure S13. Average scaled expression of the identified sex-specific gene sets in relation to the MOF and sex during the defined phases of the severity. Figure S14. Scaled expression of the identified sex-specific gene sets in the early pre-acute, pre-acute, acute and late post-acute phases of patients with burn injury. Table S1. Distribution of microarray samples across the phases. Table S2. Distribution of microarray samples across the sampling groups. Table S3. Patient characteristics. Table S4. List of significant gene sets in all investigated phases. Table S5. List of significant gene sets in the acute-phase of the propensityscore matched subset of male and female patients. (DOC 4496 kb) [file 10020_2019_87_MOESM1_ESM.doc]

**Temporal progression of gene regulation of peripheral white blood cells explains gender dimorphism of critically ill patients after trauma**

Amol Kolte, Rainer König

Integrated Research and Treatment Center, Center for Sepsis Control and Care (CSCC), Jena University Hospital, Am Klinikum 1, 07747 Jena, Germany, and

Network Modeling, Leibniz Institute for Natural Product Research and Infection Biology - Hans Knöll Institute Jena, Beutenbergstrasse 11a, 07745 Jena, Germany.

**Additional Material**

Content

[Figure S1: Workflow for selecting the patients 2](#__RefHeading___Toc7688988)

[Figure S2: Definition and distribution of unstable phases 3](#__RefHeading___Toc7688989)

[Figure S3: Comparison of the AIS scores at baseline and Marshall MOF severity profiles after synchronization 3](#__RefHeading___Toc7688990)

[Figure S4: Comparison of the day of highest severity (Marshall MOF) in male and female patients 4](#__RefHeading___Toc7688991)

[Figure S5: Complete cluster of upregulated gene sets in females in the early pre-acute phase. 5](#__RefHeading___Toc7688992)

[Figure S6: Complete cluster of upregulated gene sets in females in at least two phases among the early pre-acute, pre-acute and acute phase. 6](#__RefHeading___Toc7688993)

[Figure S7: Cluster of upregulated gene sets in female patients in the pre-acute phase. 7](#__RefHeading___Toc7688994)

[Figure S8: Cluster of upregulated gene sets in female patients during the acute phase. 8](#__RefHeading___Toc7688995)

[Figure S9: Cluster of downregulated gene sets in female patients during the acute phase. 9](#__RefHeading___Toc7688996)

[Figure S10: Cluster of downregulated gene sets in female patients in at least two phases among the early pre-acute, pre-acute and acute phase. 10](#__RefHeading___Toc7688997)

[Figure S11: Scaled expression of energy metabolism related genes in the acute phase. 11](#__RefHeading___Toc7688998)

[Figure S12: Complete cluster of upregulated gene sets in female patients in the late post-acute phase. 11](#__RefHeading___Toc7688999)

[Figure S13: The average scaled expression of the identified sex-specific gene sets in relation to the MOF and sex during the defined phases of the severity. 13](#__RefHeading___Toc7689000)

[Figure S14: Scaled expression of the identified sex-specific gene sets in the early pre-acute, pre-acute, acute and late post-acute phases of patients with burn injury. 13](#__RefHeading___Toc7689001)

[Table S1: Distribution of microarray samples across the phases. 15](#__RefHeading___Toc7689002)

[Table S2: Distribution of microarray samples across the sampling groups. 15](#__RefHeading___Toc7689003)

[Table S3: Patient characteristics. 15](#__RefHeading___Toc7689004)

[Table S4: List of significant gene sets in all investigated phases. 16](#__RefHeading___Toc7689005)

[Table S5: List of significant gene sets in the acute-phase of the propensity-score matched sub-set of male and female patients. 25](#__RefHeading___Toc7689006)

**Additional Figures**

**
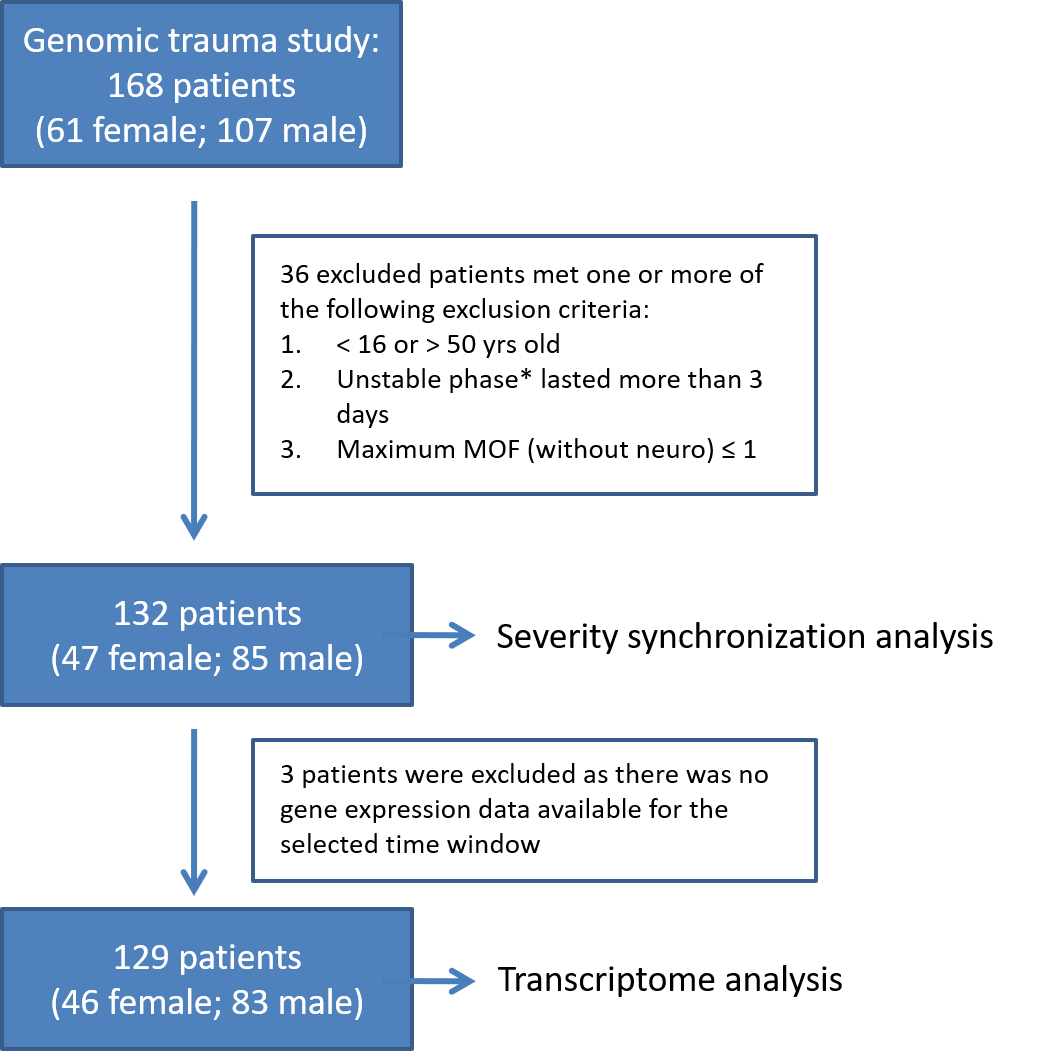
**

## Figure S1: Workflow for selecting the patients

A subset of 132 (85 male; 47 female) out of 168 patients was included in our severity synchronization analysis. The selection was made based on their age, length of the unstable phase and MOF scores until 28 days from the time point of injury. Among these, for 129 patients (83 male, 46 female) transcriptomic profiles were available matching to the investigated time window, i.e. from 3 days before the acute phase to 3 days after the acute phase. Finally, 119 transcription profiles of 46 female and 211 of 83 male patients were used for the transcriptome analysis.

**
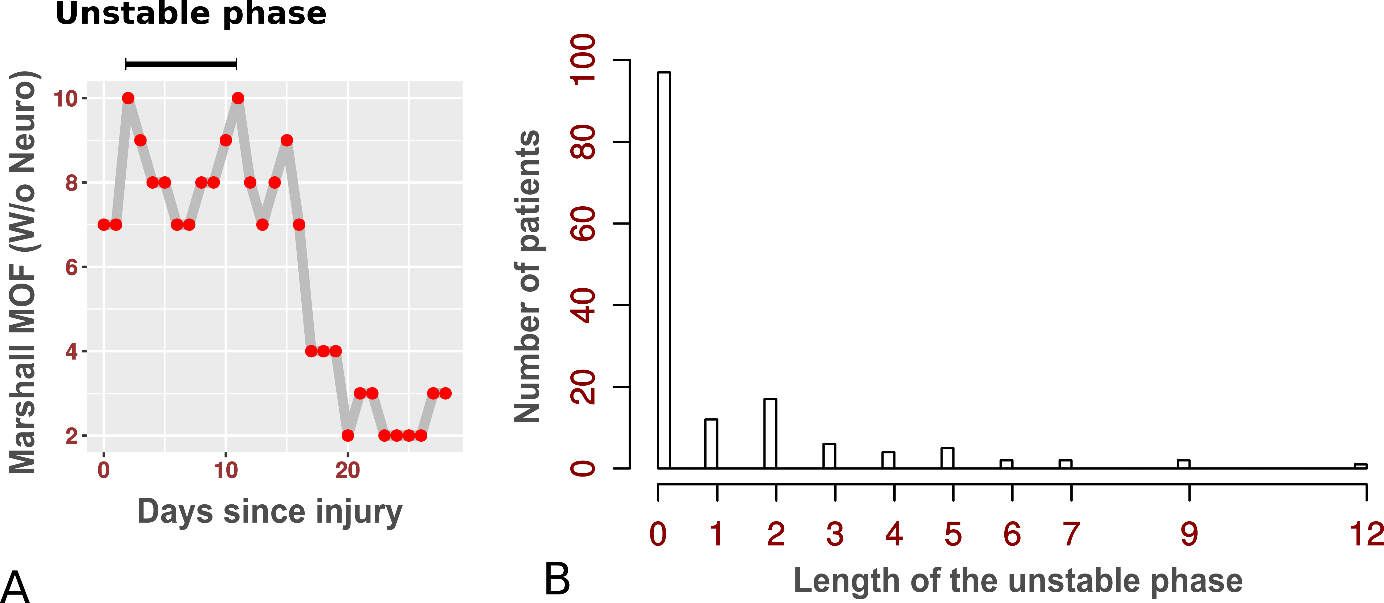
**

## Figure S2: Definition and distribution of unstable phases

A MOF score profile was defined to be a *complicated* MOF score profile if it contained two or more days with the highest MOF score. (A) An example of a complicated profile is shown here with an unstable phase of more than 3 days. An unstable phase was defined as the days between the first day of the highest MOF score and the last day of the highest MOF score. Patients were not used for our study if this unstable phase was > 3 days. (B) The distribution of the lengths of unstable phases across all patients.


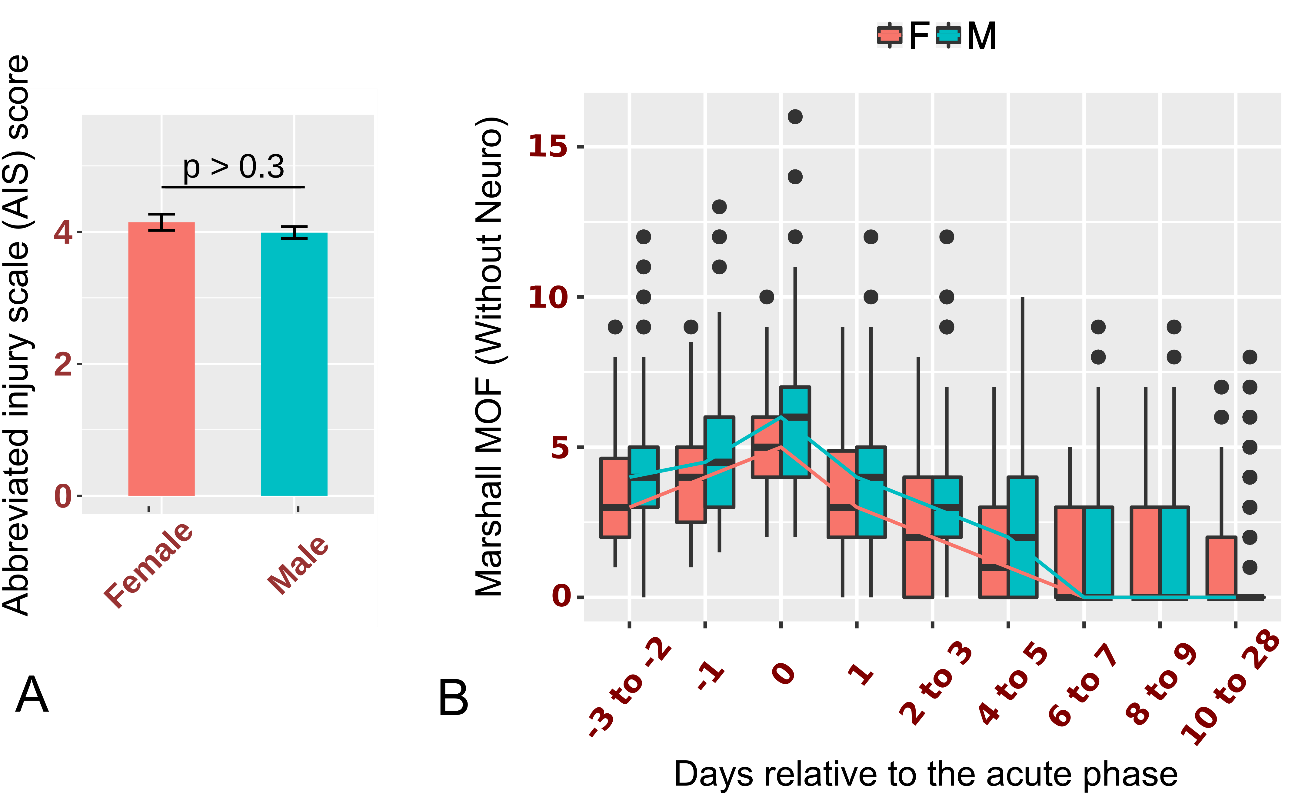


## Figure S3: Comparison of the AIS scores at baseline and Marshall MOF severity profiles after synchronization

(A) The abbreviated injury scale (AIS) score was comparable between the sexes (male patients: AIS=3.99 (95% CI: 3.81-4.17), female patients: AIS=4.15 (95% CI: 3.91-4.39; p > 0.3)) at admission. (B) The Marshall MOF score (without neurological components) of the synchronized profiles was higher in male patients at the later time points.

**
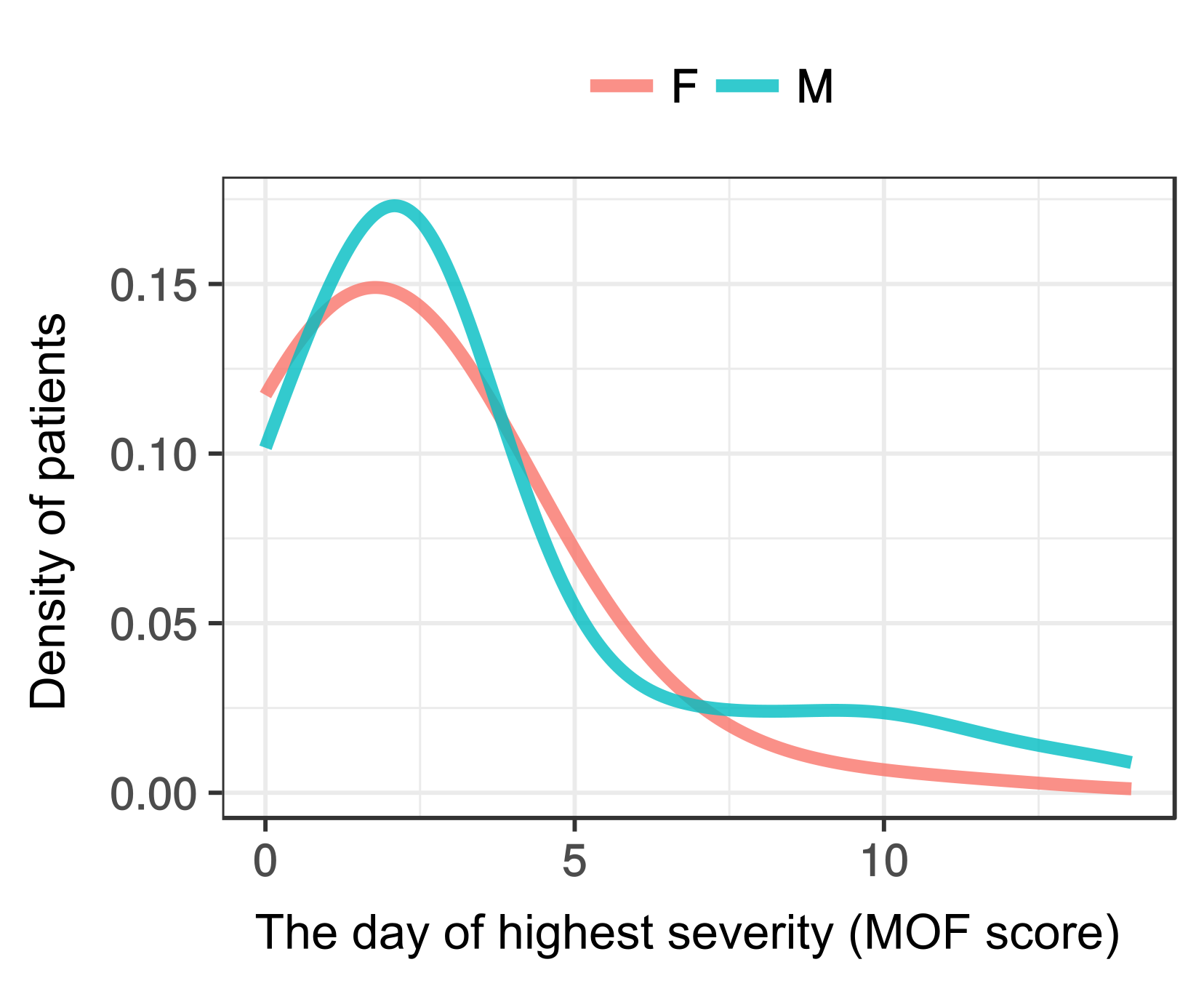
**

## Figure S4: Comparison of the day of highest severity (Marshall MOF) in male and female patients

Male and female patients showed a similar distribution of the highest MOF score after baseline. To note there was no significant (p > 0.1, Wilcoxon rank-sum test) difference in the distributions.

**
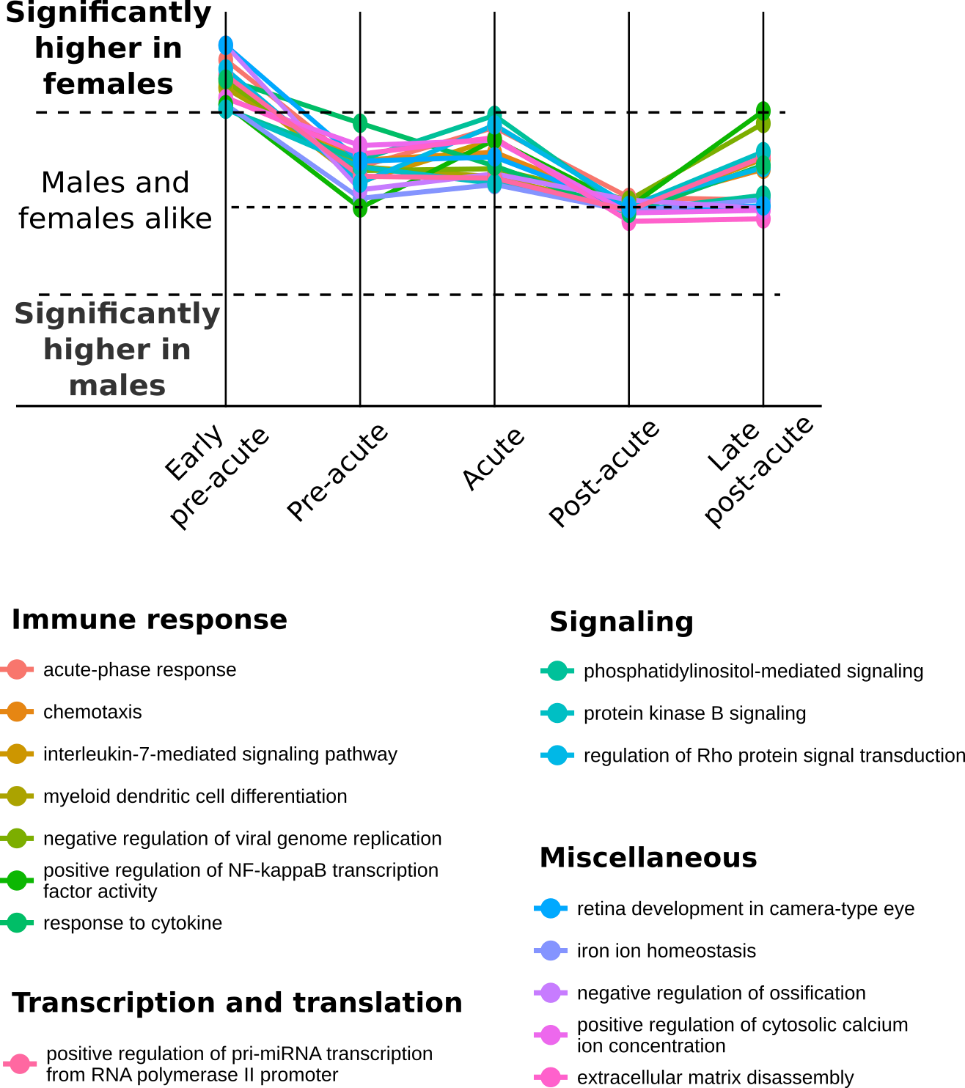
**

## Figure S5: Complete cluster of upregulated gene sets in females in the early pre-acute phase.

**
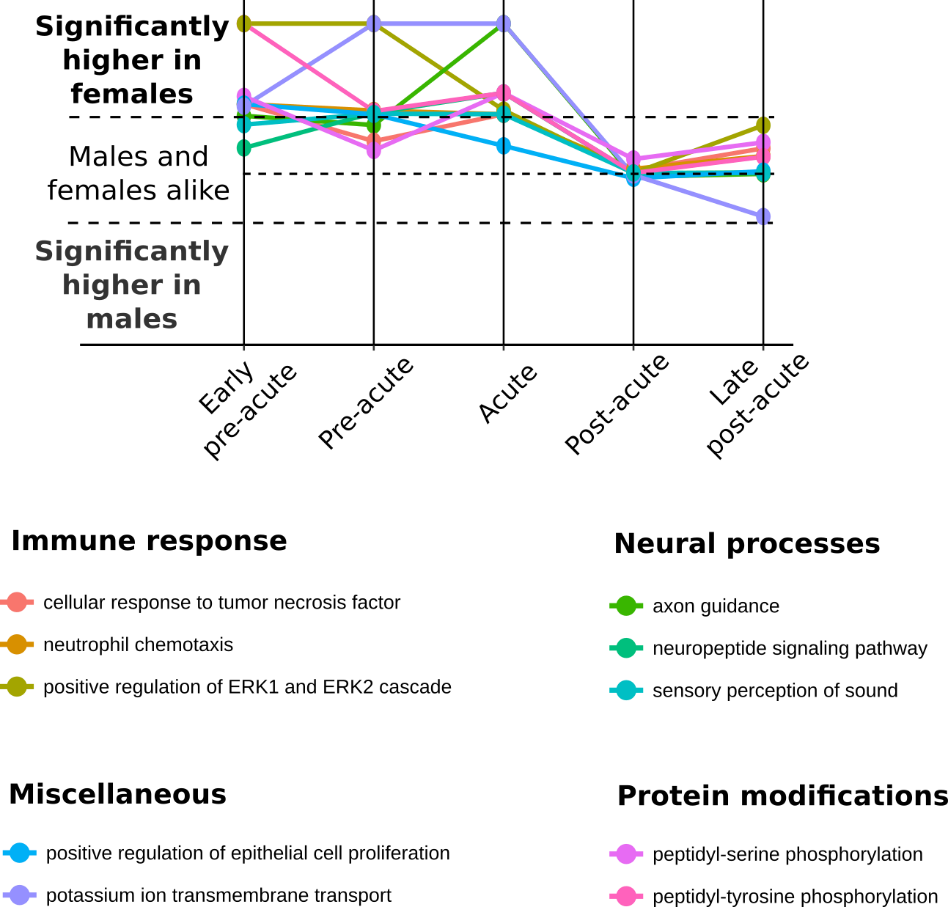
**

## Figure S6: Complete cluster of upregulated gene sets in females in at least two phases among the early pre-acute, pre-acute and acute phase.

**
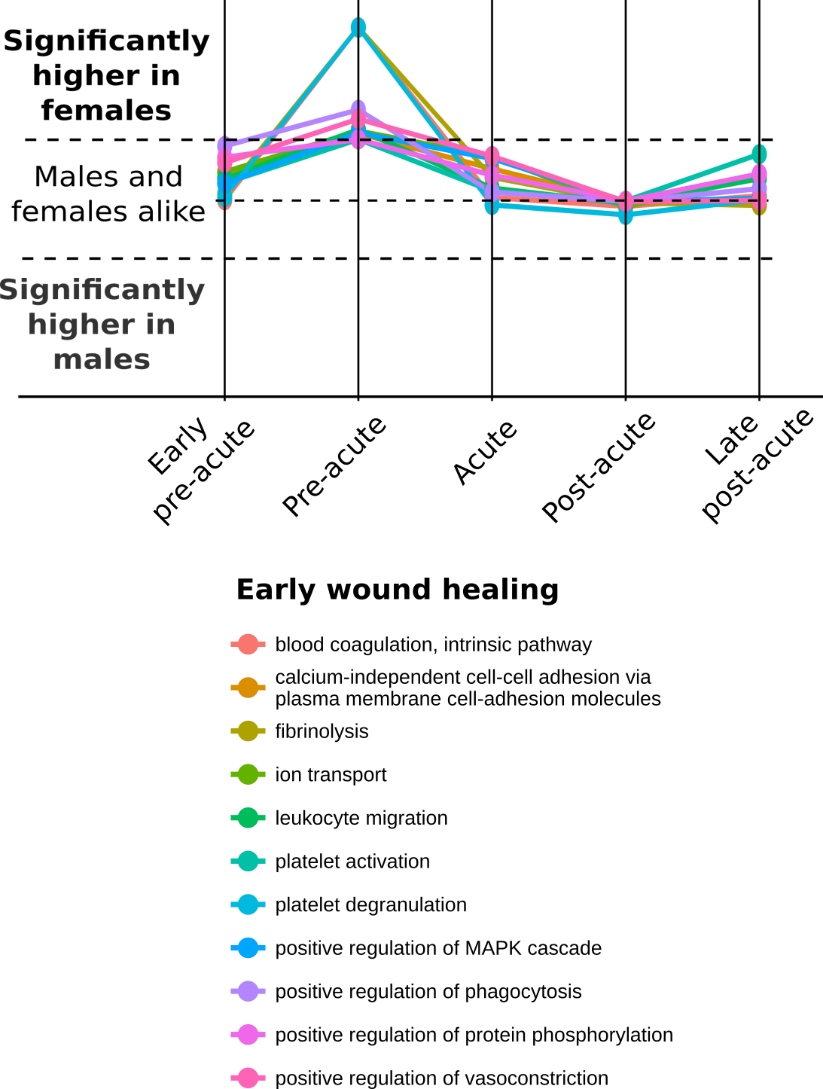
**

## Figure S7: Cluster of upregulated gene sets in female patients in the pre-acute phase.

**
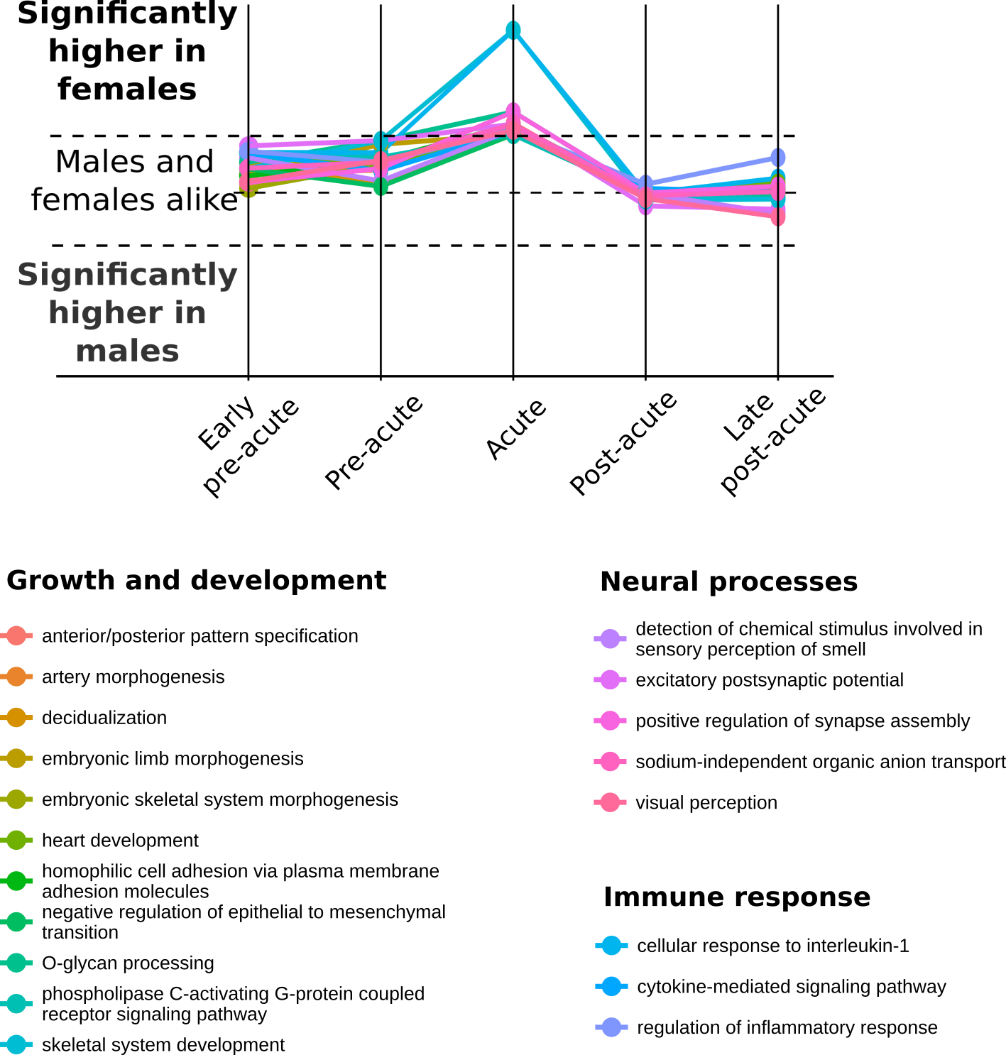
**

## Figure S8: Cluster of upregulated gene sets in female patients during the acute phase.


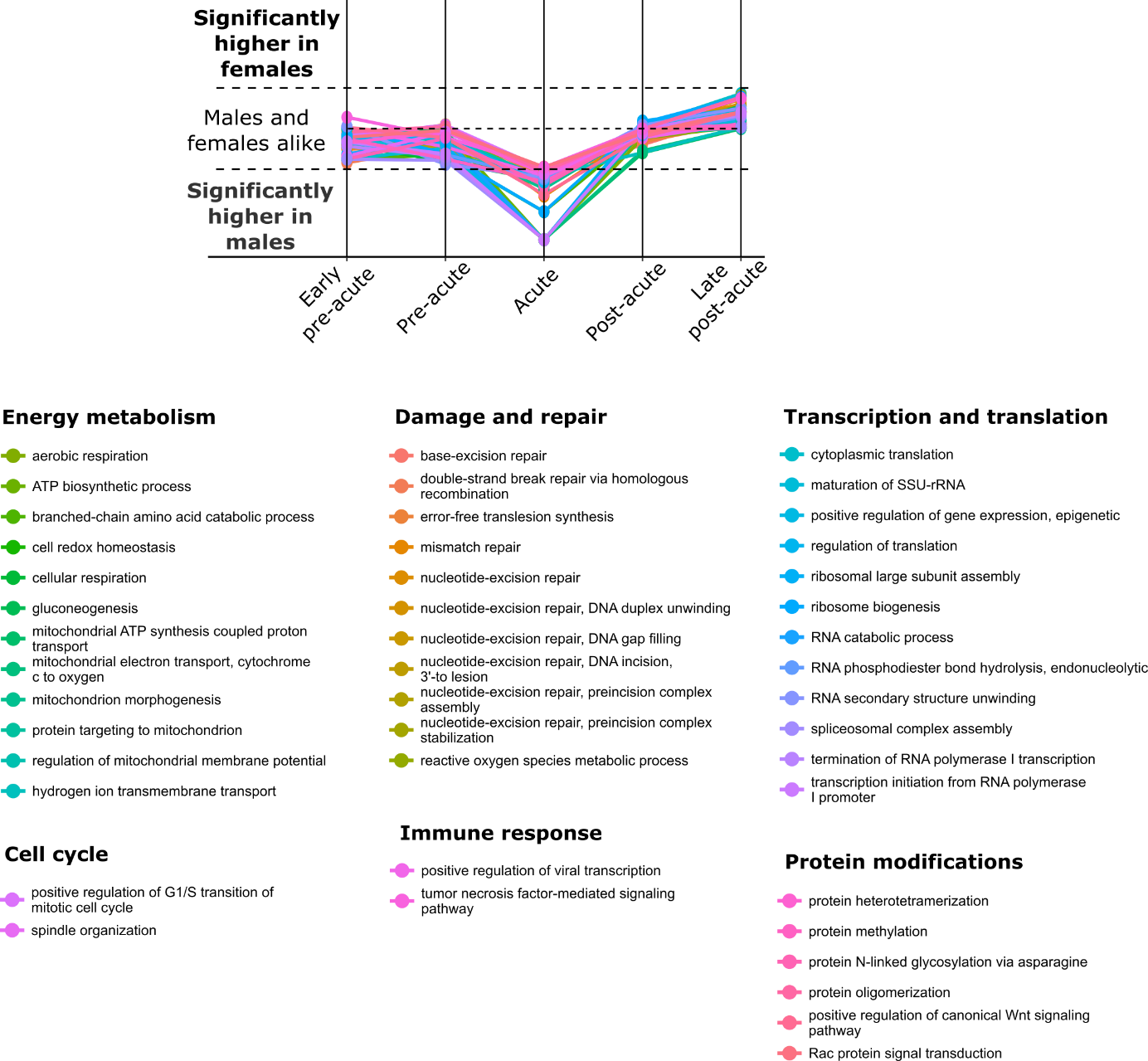


## Figure S9: Cluster of downregulated gene sets in female patients during the acute phase.


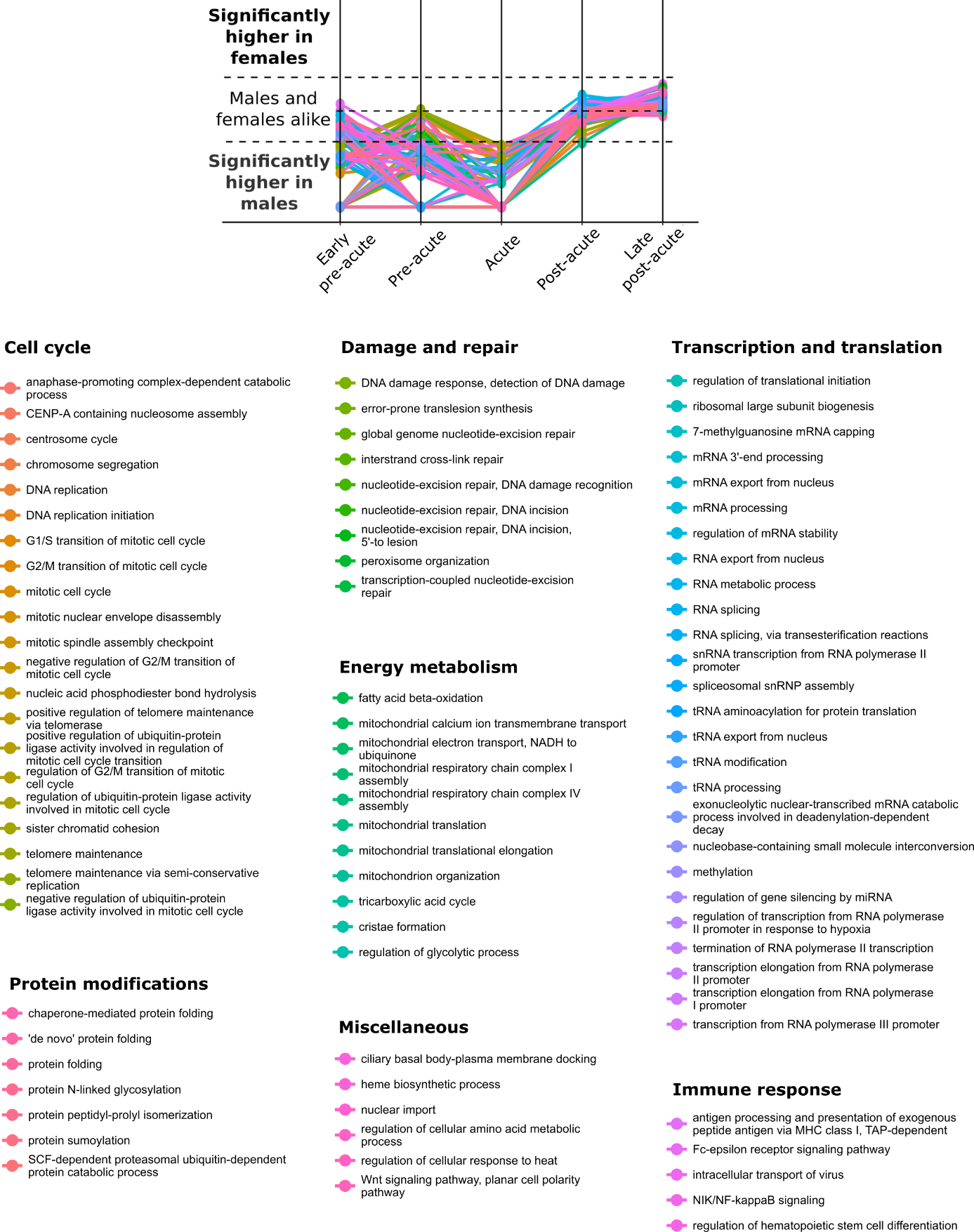


## Figure S10: Cluster of downregulated gene sets in female patients in at least two phases among the early pre-acute, pre-acute and acute phase.

**
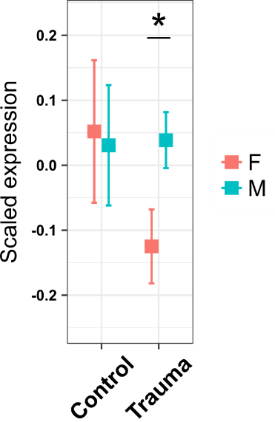
**

## Figure S11: Scaled expression of energy metabolism related genes in the acute phase.

In comparison to healthy individuals, gene sets related to energy metabolism were downregulated in female patients during the acute phase. The expression of these gene sets in male patients was comparable to healthy controls.

**
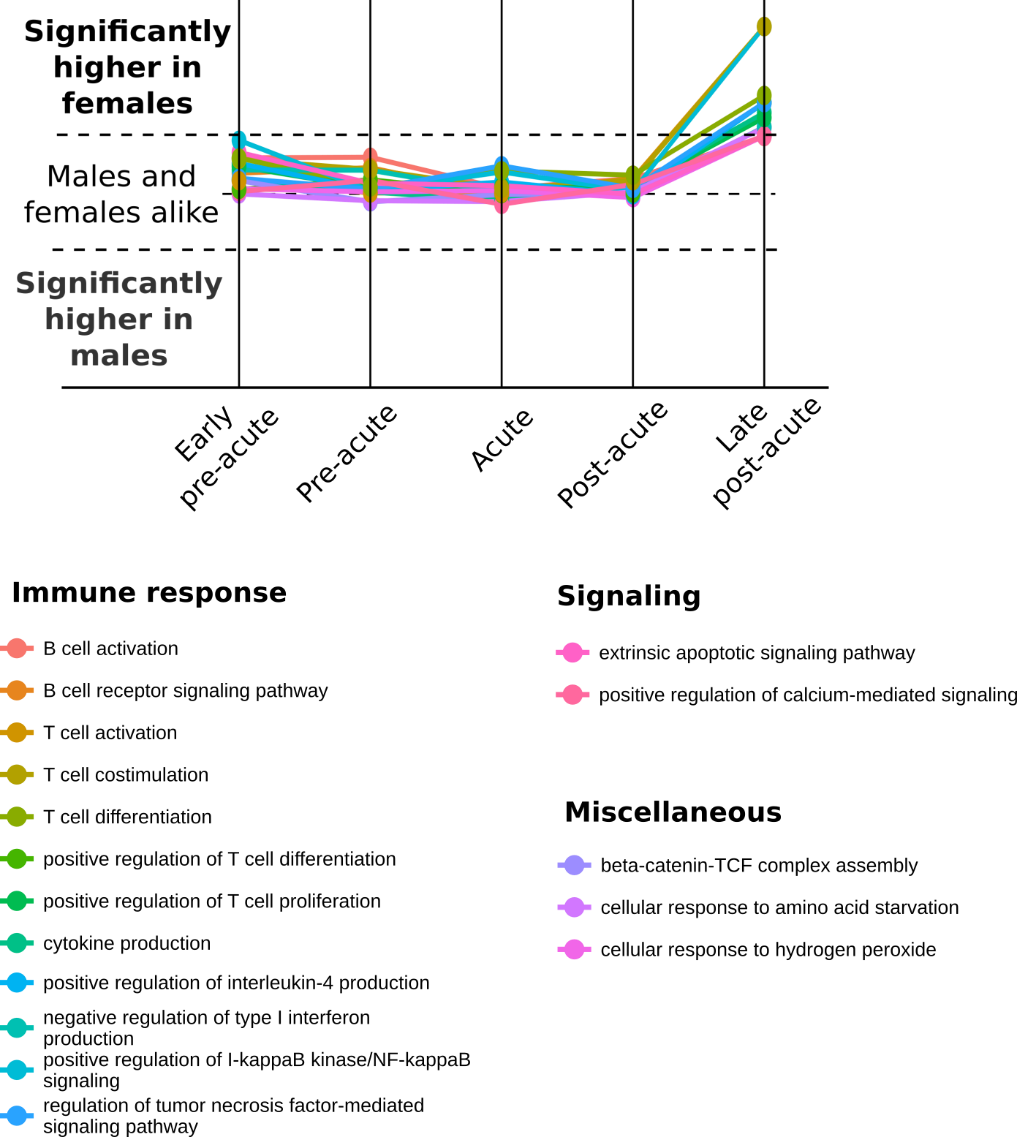
**

## Figure S12: Complete cluster of upregulated gene sets in female patients in the late post-acute phase.

**
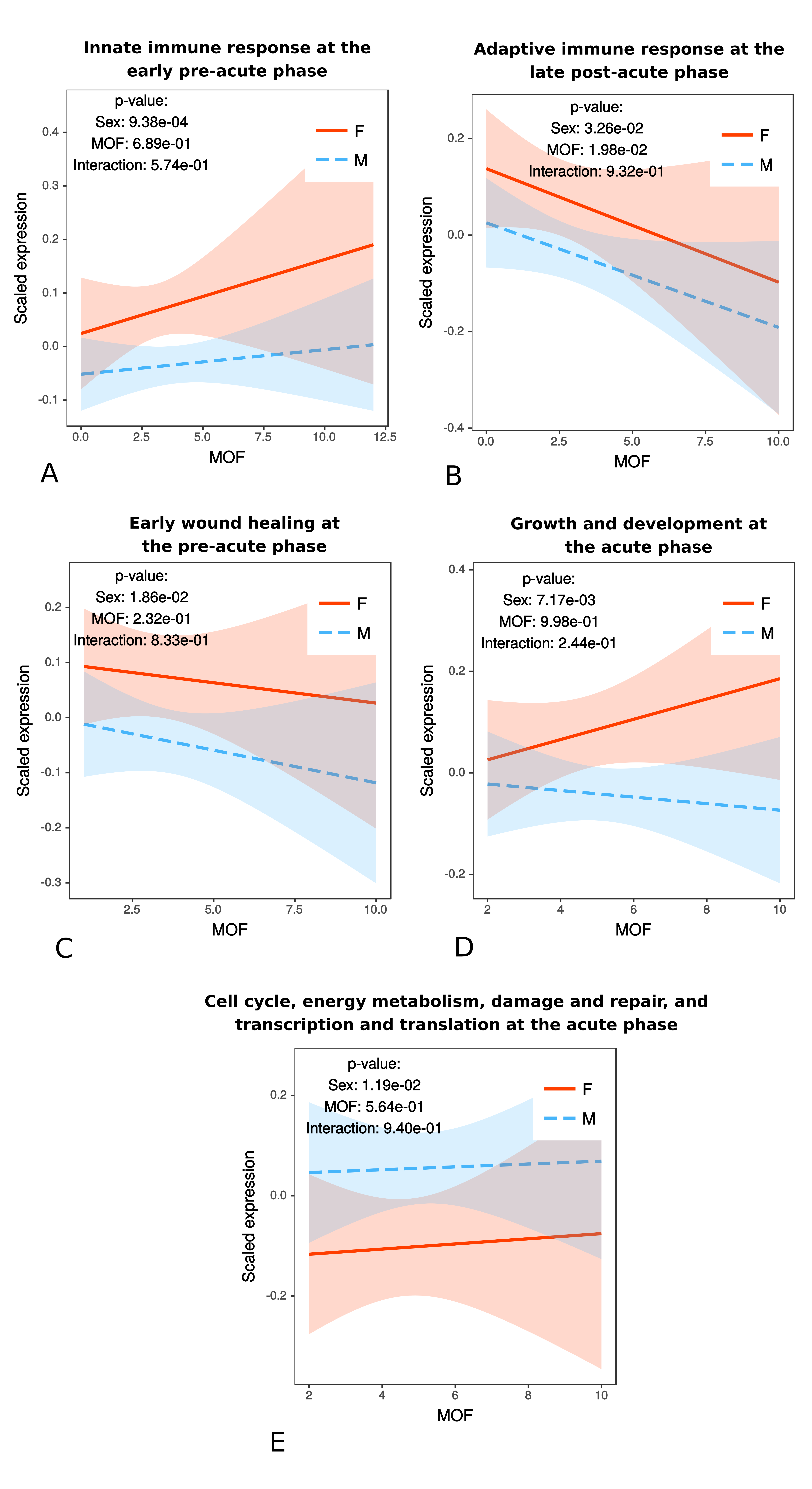
**

## Figure S13: The average scaled expression of the identified sex-specific gene sets in relation to the MOF and sex during the defined phases of the severity.

The figure depicts the results from the interaction analysis of MOF and sex of the identified gene sets of A) innate immune response in the early pre-acute phase, B) adaptive immune response in the late post-acute phase, C) early wound healing in the pre-acute phase, D) growth and development in the acute phase and, E) cell cycle, energy metabolism, damage and repair, transcription and translation in the acute phase. All the identified expression differences across phases were significantly depending on the sex variable and rather independent from the MOF scores and an interaction of sex and MOF scores.


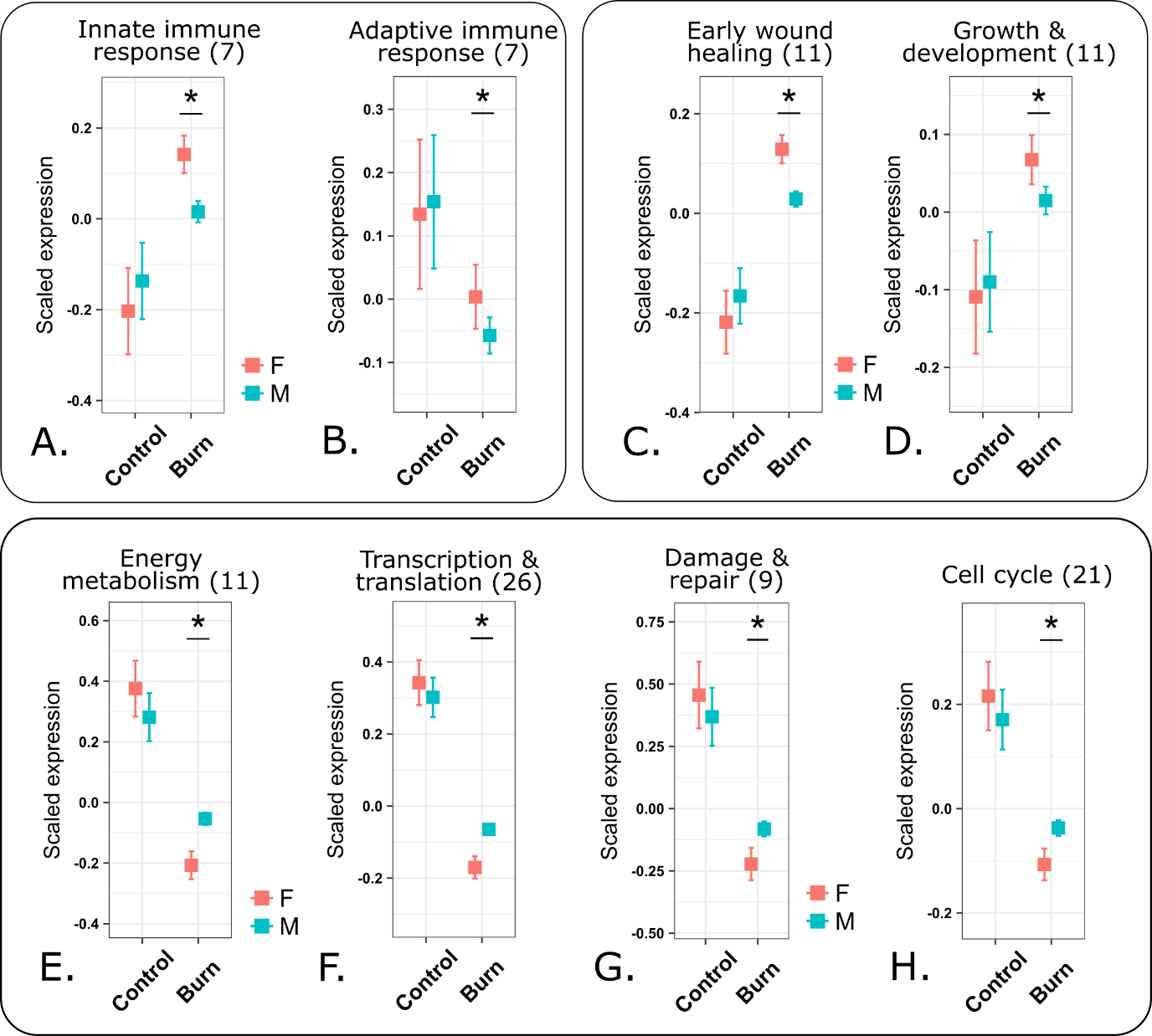


## Figure S14: Scaled expression of the identified sex-specific gene sets in the early pre-acute, pre-acute, acute and late post-acute phases of patients with burn injury.

The figure depicts the expression levels in patients with severe burn injury and healthy individuals for the identified sex-specific gene sets in the early pre-acute, pre-acute, acute and late post-acute phases of the trauma patients. The annotations of the gene sets are given on top of each panel, and the number of identified gene sets in trauma are in brackets.

**Additional Tables**

## Table S1: Distribution of microarray samples across the phases.

|  | **Early pre-acute** | **Pre-acute** | **Acute** | **Post-acute** | **Late post-acute** |
| --- | --- | --- | --- | --- | --- |
| **Male** | 63 | 29 | 52 | 21 | 46 |
| **Female** | 31 | 17 | 28 | 19 | 24 |

## Table S2: Distribution of microarray samples across the sampling groups.

|  | Male | Female | ***Row Totals*** |
| --- | --- | --- | --- |
| Group 1 | 45 | 36 | 81 |
| Group 2 | 46 | 22 | 68 |
| Group 3 | 77 | 32 | 109 |
| Group 4 | 43 | 29 | 72 |
| ***Column Totals*** | 211 | 119 | **330**  **(Grand Total)** |

## Table S3: Patient characteristics.

|  | Male (n=83) | Female (n=46) |
| --- | --- | --- |
| Age (years), mean [95% Confidence Interval] | 32.7 [30.5-34.9] | 32.7 [29.9-35.4] |
| Race, n (%) |  | |
| - African American | 7 (9%) | 2 (4%) |
| - Asian | 2 (2%) | 4 (9%) |
| - White | 72 (87%) | 39 (85%) |
| - Other | 2 (2%) | 1 (2%) |
| AIS score | 4.0 [3.8-4.2] | 4.2 [3.9-4.4] |
| Marshall MOF score (Highest) | 5.9 [5.3-6.6] | 4.8 [4.2-5.4] |
| APACHE II score | 27.1 [25.9-28.3] | 26.9 [25.2-28.6] |
| Days in ICU | 11.6 [9.7-13.5] | 11.7 [8.9-14.4] |
| Days on Ventilator | 8.9 [7.3-10.4] | 8.6 [6.1-11.1] |

## Table S4: List of significant gene sets in all investigated phases.

***Early pre-acute***

| **Early pre-acute (Up)** | | **Early pre-acute (Down)** | |
| --- | --- | --- | --- |
| transmembrane receptor protein tyrosine kinase signaling pathway | 2.00E-05 | mitotic sister chromatid segregation | 2.00E-05 |
| response to virus | 2.00E-05 | G1/S transition of mitotic cell cycle | 2.00E-05 |
| peptidyl-tyrosine phosphorylation | 2.00E-05 | G2/M transition of mitotic cell cycle | 2.00E-05 |
| peptidyl-tyrosine autophosphorylation | 2.00E-05 | mitotic cell cycle | 2.00E-05 |
| protein autophosphorylation | 2.00E-05 | DNA replication | 2.00E-05 |
| interferon-gamma-mediated signaling pathway | 2.00E-05 | DNA replication initiation | 2.00E-05 |
| type I interferon signaling pathway | 2.00E-05 | transcription-coupled nucleotide-excision repair | 2.00E-05 |
| positive regulation of ERK1 and ERK2 cascade | 2.00E-05 | translation | 2.00E-05 |
| cellular response to lipopolysaccharide | 2.00E-05 | tRNA aminoacylation for protein translation | 2.00E-05 |
| negative regulation of ossification | 2.30E-03 | protein folding | 2.00E-05 |
| retina development in camera-type eye | 2.30E-03 | 'de novo' protein folding | 2.00E-05 |
| acute-phase response | 3.93E-03 | mitotic spindle organization | 2.00E-05 |
| peptidyl-serine phosphorylation | 3.93E-03 | chromosome segregation | 2.00E-05 |
| regulation of Rho protein signal transduction | 5.51E-03 | sister chromatid cohesion | 2.00E-05 |
| neutrophil chemotaxis | 6.89E-03 | tRNA processing | 2.00E-05 |
| lipopolysaccharide-mediated signaling pathway | 6.89E-03 | regulation of G2/M transition of mitotic cell cycle | 2.00E-05 |
| positive regulation of epithelial cell proliferation | 6.89E-03 | nucleobase-containing small molecule interconversion | 2.00E-05 |
| cellular response to tumor necrosis factor | 6.89E-03 | anaphase-promoting complex-dependent catabolic process | 2.00E-05 |
| positive regulation of pri-miRNA transcription from RNA polymerase II promoter | 6.89E-03 | methylation | 2.00E-05 |
| interleukin-7-mediated signaling pathway | 7.87E-03 | mitochondrial translation | 2.00E-05 |
| response to cytokine | 8.03E-03 | CENP-A containing nucleosome assembly | 2.00E-05 |
| regulation of immune response | 8.03E-03 | negative regulation of ubiquitin-protein ligase activity involved in mitotic cell cycle | 2.00E-05 |
| potassium ion transmembrane transport | 8.03E-03 | positive regulation of ubiquitin-protein ligase activity involved in regulation of mitotic cell cycle transition | 2.00E-05 |
| negative regulation of viral genome replication | 8.81E-03 | regulation of ubiquitin-protein ligase activity involved in mitotic cell cycle | 2.00E-05 |
| chemotaxis | 1.06E-02 | chaperone-mediated protein folding | 2.00E-05 |
| myeloid dendritic cell differentiation | 1.12E-02 | mitochondrial translational elongation | 2.00E-05 |
| extracellular matrix disassembly | 1.57E-02 | mitochondrial translational termination | 2.00E-05 |
| axon guidance | 1.61E-02 | translational initiation | 9.18E-04 |
| positive regulation of cytosolic calcium ion concentration | 1.65E-02 | heme biosynthetic process | 9.18E-04 |
| iron ion homeostasis | 2.04E-02 | mitotic nuclear envelope disassembly | 9.18E-04 |
| positive regulation of NF-kappaB transcription factor activity | 2.07E-02 | interstrand cross-link repair | 1.72E-03 |
| protein kinase B signaling | 2.43E-02 | cristae formation | 1.72E-03 |
| phosphatidylinositol-mediated signaling | 2.43E-02 | error-prone translesion synthesis | 2.50E-03 |
|  |  | mitochondrial respiratory chain complex I assembly | 3.24E-03 |
|  |  | nucleotide-excision repair, DNA incision, 5'-to lesion | 3.83E-03 |
|  |  | snRNA transcription from RNA polymerase II promoter | 3.83E-03 |
|  |  | spliceosomal snRNP assembly | 4.35E-03 |
|  |  | DNA damage response, detection of DNA damage | 4.35E-03 |
|  |  | transcription from RNA polymerase III promoter | 4.94E-03 |
|  |  | protein N-linked glycosylation | 5.25E-03 |
|  |  | mitochondrion organization | 5.25E-03 |
|  |  | ciliary basal body-plasma membrane docking | 5.25E-03 |
|  |  | nucleotide-excision repair, DNA incision | 5.39E-03 |
|  |  | protein ubiquitination involved in ubiquitin-dependent protein catabolic process | 5.39E-03 |
|  |  | nuclear import | 5.39E-03 |
|  |  | regulation of cellular response to heat | 5.39E-03 |
|  |  | tricarboxylic acid cycle | 6.31E-03 |
|  |  | tRNA export from nucleus | 6.31E-03 |
|  |  | centrosome cycle | 7.16E-03 |
|  |  | mitotic spindle assembly | 7.16E-03 |
|  |  | protein sumoylation | 7.56E-03 |
|  |  | regulation of transcription involved in G1/S transition of mitotic cell cycle | 7.79E-03 |
|  |  | regulation of signal transduction by p53 class mediator | 7.79E-03 |
|  |  | mRNA export from nucleus | 1.17E-02 |
|  |  | nucleic acid phosphodiester bond hydrolysis | 1.50E-02 |
|  |  | viral transcription | 1.52E-02 |
|  |  | mitotic spindle assembly checkpoint | 1.76E-02 |
|  |  | regulation of gene silencing by miRNA | 1.76E-02 |
|  |  | global genome nucleotide-excision repair | 1.82E-02 |
|  |  | mitotic cytokinesis | 1.84E-02 |
|  |  | nucleotide-excision repair, DNA damage recognition | 1.88E-02 |
|  |  | telomere maintenance via semi-conservative replication | 1.88E-02 |
|  |  | cellular response to UV | 1.88E-02 |
|  |  | mitochondrial calcium ion transmembrane transport | 2.07E-02 |
|  |  | telomere maintenance | 2.42E-02 |

***Pre-acute***

| **Pre-acute (Up)** | | **Pre-acute (Down)** | |
| --- | --- | --- | --- |
| platelet degranulation | 2.00E-05 | regulation of glycolytic process | 2.00E-05 |
| blood coagulation, intrinsic pathway | 2.00E-05 | transcription-coupled nucleotide-excision repair | 2.00E-05 |
| fibrinolysis | 2.00E-05 | transcription elongation from RNA polymerase II promoter | 2.00E-05 |
| type I interferon signaling pathway | 2.00E-05 | termination of RNA polymerase II transcription | 2.00E-05 |
| positive regulation of ERK1 and ERK2 cascade | 2.00E-05 | mRNA processing | 2.00E-05 |
| potassium ion transmembrane transport | 2.00E-05 | mRNA export from nucleus | 2.00E-05 |
| protein autophosphorylation | 3.44E-03 | tRNA export from nucleus | 2.00E-05 |
| positive regulation of phagocytosis | 3.44E-03 | translation | 2.00E-05 |
| positive regulation of vasoconstriction | 6.12E-03 | translational initiation | 2.00E-05 |
| neutrophil chemotaxis | 1.10E-02 | tRNA aminoacylation for protein translation | 2.00E-05 |
| adaptive immune response | 1.15E-02 | protein folding | 2.00E-05 |
| peptidyl-tyrosine phosphorylation | 1.15E-02 | regulation of cellular amino acid metabolic process | 2.00E-05 |
| leukocyte migration | 1.18E-02 | SRP-dependent cotranslational protein targeting to membrane | 2.00E-05 |
| interferon-gamma-mediated signaling pathway | 1.18E-02 | fatty acid beta-oxidation | 2.00E-05 |
| regulation of immune response | 1.29E-02 | RNA splicing | 2.00E-05 |
| ion transport | 1.30E-02 | negative regulation of G2/M transition of mitotic cell cycle | 2.00E-05 |
| neuropeptide signaling pathway | 1.30E-02 | viral transcription | 2.00E-05 |
| sensory perception of sound | 1.38E-02 | mRNA 3'-end processing | 2.00E-05 |
| transmembrane receptor protein tyrosine kinase signaling pathway | 1.44E-02 | anaphase-promoting complex-dependent catabolic process | 2.00E-05 |
| positive regulation of MAPK cascade | 1.44E-02 | SCF-dependent proteasomal ubiquitin-dependent protein catabolic process | 2.00E-05 |
| positive regulation of epithelial cell proliferation | 1.44E-02 | mitochondrial respiratory chain complex I assembly | 2.00E-05 |
| calcium-independent cell-cell adhesion via plasma membrane cell-adhesion molecules | 1.50E-02 | mitochondrial respiratory chain complex IV assembly | 2.00E-05 |
| positive regulation of protein phosphorylation | 2.18E-02 | snRNA transcription from RNA polymerase II promoter | 2.00E-05 |
| platelet activation | 2.18E-02 | regulation of mRNA stability | 2.00E-05 |
|  |  | negative regulation of ubiquitin-protein ligase activity involved in mitotic cell cycle | 2.00E-05 |
|  |  | positive regulation of ubiquitin-protein ligase activity involved in regulation of mitotic cell cycle transition | 2.00E-05 |
|  |  | regulation of gene silencing by miRNA | 2.00E-05 |
|  |  | chaperone-mediated protein folding | 2.00E-05 |
|  |  | mitochondrial translational elongation | 2.00E-05 |
|  |  | mitochondrial translational termination | 2.00E-05 |
|  |  | intracellular transport of virus | 2.00E-05 |
|  |  | maturation of SSU-rRNA from tricistronic rRNA transcript (SSU-rRNA, 5.8S rRNA, LSU-rRNA) | 7.25E-04 |
|  |  | RNA export from nucleus | 7.25E-04 |
|  |  | ubiquitin-dependent ERAD pathway | 7.25E-04 |
|  |  | Fc-epsilon receptor signaling pathway | 7.25E-04 |
|  |  | exonucleolytic nuclear-transcribed mRNA catabolic process involved in deadenylation-dependent decay | 7.25E-04 |
|  |  | regulation of transcription from RNA polymerase II promoter in response to hypoxia | 7.25E-04 |
|  |  | regulation of cellular response to heat | 7.25E-04 |
|  |  | regulation of G2/M transition of mitotic cell cycle | 1.28E-03 |
|  |  | NIK/NF-kappaB signaling | 1.28E-03 |
|  |  | T cell receptor signaling pathway | 1.28E-03 |
|  |  | Wnt signaling pathway, planar cell polarity pathway | 1.28E-03 |
|  |  | regulation of hematopoietic stem cell differentiation | 1.28E-03 |
|  |  | G2/M transition of mitotic cell cycle | 1.76E-03 |
|  |  | RNA splicing, via transesterification reactions | 1.76E-03 |
|  |  | ubiquitin-dependent protein catabolic process | 1.76E-03 |
|  |  | stem cell population maintenance | 1.76E-03 |
|  |  | tricarboxylic acid cycle | 2.16E-03 |
|  |  | mitotic nuclear envelope disassembly | 2.16E-03 |
|  |  | RNA metabolic process | 2.16E-03 |
|  |  | tRNA methylation | 2.16E-03 |
|  |  | transcription from RNA polymerase III promoter | 2.50E-03 |
|  |  | Golgi organization | 2.50E-03 |
|  |  | mitochondrial translation | 2.50E-03 |
|  |  | COPII vesicle coating | 2.50E-03 |
|  |  | tRNA modification | 3.44E-03 |
|  |  | sister chromatid cohesion | 3.80E-03 |
|  |  | ribosomal small subunit biogenesis | 3.80E-03 |
|  |  | 'de novo' protein folding | 4.13E-03 |
|  |  | protein sumoylation | 4.13E-03 |
|  |  | mitochondrial electron transport, NADH to ubiquinone | 5.87E-03 |
|  |  | peroxisome organization | 8.00E-03 |
|  |  | protein localization to centrosome | 8.74E-03 |
|  |  | spliceosomal snRNP assembly | 9.04E-03 |
|  |  | intra-Golgi vesicle-mediated transport | 9.18E-03 |
|  |  | ribosomal large subunit biogenesis | 9.18E-03 |
|  |  | protein peptidyl-prolyl isomerization | 9.87E-03 |
|  |  | mitotic cell cycle | 9.98E-03 |
|  |  | Golgi to plasma membrane transport | 9.98E-03 |
|  |  | mRNA polyadenylation | 1.02E-02 |
|  |  | RNA phosphodiester bond hydrolysis, exonucleolytic | 1.16E-02 |
|  |  | tRNA processing | 1.22E-02 |
|  |  | 7-methylguanosine mRNA capping | 1.25E-02 |
|  |  | antigen processing and presentation of exogenous peptide antigen via MHC class I, TAP-dependent | 1.38E-02 |
|  |  | methylation | 1.40E-02 |
|  |  | response to unfolded protein | 1.49E-02 |
|  |  | positive regulation of telomere maintenance via telomerase | 1.54E-02 |
|  |  | RNA processing | 1.59E-02 |
|  |  | nuclear-transcribed mRNA catabolic process, nonsense-mediated decay | 1.60E-02 |
|  |  | ciliary basal body-plasma membrane docking | 1.82E-02 |
|  |  | transcription initiation from RNA polymerase II promoter | 1.87E-02 |
|  |  | regulation of translational initiation | 1.99E-02 |
|  |  | global genome nucleotide-excision repair | 1.99E-02 |
|  |  | transcription elongation from RNA polymerase I promoter | 2.33E-02 |

***Acute***

| **Acute (Up)** | | **Acute (Down)** | |
| --- | --- | --- | --- |
| skeletal system development | 2.00E-05 | ribosomal large subunit assembly | 2.00E-05 |
| axon guidance | 2.00E-05 | G1/S transition of mitotic cell cycle | 2.00E-05 |
| type I interferon signaling pathway | 2.00E-05 | nuclear-transcribed mRNA catabolic process, nonsense-mediated decay | 2.00E-05 |
| cellular response to interleukin-1 | 2.00E-05 | spliceosomal complex assembly | 2.00E-05 |
| potassium ion transmembrane transport | 2.00E-05 | spliceosomal snRNP assembly | 2.00E-05 |
| neuropeptide signaling pathway | 3.06E-03 | maturation of SSU-rRNA from tricistronic rRNA transcript (SSU-rRNA, 5.8S rRNA, LSU-rRNA) | 2.00E-05 |
| peptidyl-serine phosphorylation | 3.06E-03 | nucleotide-excision repair, DNA damage recognition | 2.00E-05 |
| peptidyl-tyrosine phosphorylation | 3.06E-03 | nucleotide-excision repair, DNA duplex unwinding | 2.00E-05 |
| regulation of ion transmembrane transport | 3.06E-03 | cytoplasmic translation | 2.00E-05 |
| O-glycan processing | 4.59E-03 | antigen processing and presentation of exogenous peptide antigen via MHC class I, TAP-dependent | 2.00E-05 |
| protein autophosphorylation | 4.59E-03 | mitochondrial electron transport, NADH to ubiquinone | 2.00E-05 |
| positive regulation of synapse assembly | 4.59E-03 | mitochondrial electron transport, cytochrome c to oxygen | 2.00E-05 |
| transmembrane receptor protein tyrosine kinase signaling pathway | 6.36E-03 | DNA replication | 2.00E-05 |
| artery morphogenesis | 9.84E-03 | transcription-coupled nucleotide-excision repair | 2.00E-05 |
| sodium-independent organic anion transport | 1.03E-02 | nucleotide-excision repair, preincision complex stabilization | 2.00E-05 |
| excitatory postsynaptic potential | 1.03E-02 | nucleotide-excision repair, preincision complex assembly | 2.00E-05 |
| homophilic cell adhesion via plasma membrane adhesion molecules | 1.07E-02 | nucleotide-excision repair, DNA incision, 3'-to lesion | 2.00E-05 |
| positive regulation of ERK1 and ERK2 cascade | 1.07E-02 | nucleotide-excision repair, DNA incision, 5'-to lesion | 2.00E-05 |
| sensory perception of sound | 1.44E-02 | transcription initiation from RNA polymerase I promoter | 2.00E-05 |
| neutrophil chemotaxis | 1.44E-02 | transcription elongation from RNA polymerase I promoter | 2.00E-05 |
| embryonic skeletal system morphogenesis | 1.44E-02 | termination of RNA polymerase I transcription | 2.00E-05 |
| interferon-gamma-mediated signaling pathway | 1.44E-02 | transcription elongation from RNA polymerase II promoter | 2.00E-05 |
| cellular response to tumor necrosis factor | 1.44E-02 | termination of RNA polymerase II transcription | 2.00E-05 |
| decidualization | 1.48E-02 | 7-methylguanosine mRNA capping | 2.00E-05 |
| regulation of inflammatory response | 1.48E-02 | transcription from RNA polymerase III promoter | 2.00E-05 |
| detection of chemical stimulus involved in sensory perception of smell | 1.48E-02 | RNA catabolic process | 2.00E-05 |
| potassium ion transport | 1.53E-02 | mRNA export from nucleus | 2.00E-05 |
| visual perception | 1.57E-02 | tRNA export from nucleus | 2.00E-05 |
| anterior/posterior pattern specification | 1.61E-02 | translation | 2.00E-05 |
| cytokine-mediated signaling pathway | 1.65E-02 | translational initiation | 2.00E-05 |
| embryonic limb morphogenesis | 2.04E-02 | tRNA aminoacylation for protein translation | 2.00E-05 |
| negative regulation of epithelial to mesenchymal transition | 2.07E-02 | regulation of translational initiation | 2.00E-05 |
| phospholipase C-activating G-protein coupled receptor signaling pathway | 2.09E-02 | protein folding | 2.00E-05 |
| heart development | 2.11E-02 | 'de novo' protein folding | 2.00E-05 |
|  |  | regulation of cellular amino acid metabolic process | 2.00E-05 |
|  |  | SRP-dependent cotranslational protein targeting to membrane | 2.00E-05 |
|  |  | protein targeting to mitochondrion | 2.00E-05 |
|  |  | fatty acid beta-oxidation | 2.00E-05 |
|  |  | ATP biosynthetic process | 2.00E-05 |
|  |  | heme biosynthetic process | 2.00E-05 |
|  |  | mitochondrion organization | 2.00E-05 |
|  |  | sister chromatid cohesion | 2.00E-05 |
|  |  | tRNA processing | 2.00E-05 |
|  |  | RNA splicing | 2.00E-05 |
|  |  | aerobic respiration | 2.00E-05 |
|  |  | regulation of G2/M transition of mitotic cell cycle | 2.00E-05 |
|  |  | negative regulation of G2/M transition of mitotic cell cycle | 2.00E-05 |
|  |  | RNA metabolic process | 2.00E-05 |
|  |  | protein sumoylation | 2.00E-05 |
|  |  | viral transcription | 2.00E-05 |
|  |  | maturation of SSU-rRNA | 2.00E-05 |
|  |  | anaphase-promoting complex-dependent catabolic process | 2.00E-05 |
|  |  | SCF-dependent proteasomal ubiquitin-dependent protein catabolic process | 2.00E-05 |
|  |  | mitochondrial translation | 2.00E-05 |
|  |  | mitochondrial respiratory chain complex I assembly | 2.00E-05 |
|  |  | mitochondrial respiratory chain complex IV assembly | 2.00E-05 |
|  |  | nucleotide-excision repair, DNA incision | 2.00E-05 |
|  |  | NIK/NF-kappaB signaling | 2.00E-05 |
|  |  | ribosomal large subunit biogenesis | 2.00E-05 |
|  |  | ribosomal small subunit biogenesis | 2.00E-05 |
|  |  | error-prone translesion synthesis | 2.00E-05 |
|  |  | cristae formation | 2.00E-05 |
|  |  | DNA damage response, detection of DNA damage | 2.00E-05 |
|  |  | mitochondrial ATP synthesis coupled proton transport | 2.00E-05 |
|  |  | snRNA transcription from RNA polymerase II promoter | 2.00E-05 |
|  |  | regulation of mRNA stability | 2.00E-05 |
|  |  | exonucleolytic nuclear-transcribed mRNA catabolic process involved in deadenylation-dependent decay | 2.00E-05 |
|  |  | nuclear import | 2.00E-05 |
|  |  | negative regulation of ubiquitin-protein ligase activity involved in mitotic cell cycle | 2.00E-05 |
|  |  | positive regulation of ubiquitin-protein ligase activity involved in regulation of mitotic cell cycle transition | 2.00E-05 |
|  |  | regulation of ubiquitin-protein ligase activity involved in mitotic cell cycle | 2.00E-05 |
|  |  | Wnt signaling pathway, planar cell polarity pathway | 2.00E-05 |
|  |  | regulation of gene silencing by miRNA | 2.00E-05 |
|  |  | chaperone-mediated protein folding | 2.00E-05 |
|  |  | regulation of transcription from RNA polymerase II promoter in response to hypoxia | 2.00E-05 |
|  |  | mitochondrial translational elongation | 2.00E-05 |
|  |  | mitochondrial translational termination | 2.00E-05 |
|  |  | global genome nucleotide-excision repair | 2.00E-05 |
|  |  | regulation of cellular response to heat | 2.00E-05 |
|  |  | regulation of hematopoietic stem cell differentiation | 2.00E-05 |
|  |  | mitotic cell cycle | 3.06E-04 |
|  |  | regulation of glycolytic process | 3.06E-04 |
|  |  | DNA recombination | 3.06E-04 |
|  |  | mitochondrial calcium ion transmembrane transport | 3.06E-04 |
|  |  | mitotic nuclear envelope disassembly | 3.06E-04 |
|  |  | branched-chain amino acid catabolic process | 3.06E-04 |
|  |  | methylation | 3.06E-04 |
|  |  | Fc-epsilon receptor signaling pathway | 3.06E-04 |
|  |  | ribosome biogenesis | 3.06E-04 |
|  |  | regulation of signal transduction by p53 class mediator | 3.06E-04 |
|  |  | nucleobase-containing small molecule interconversion | 5.86E-04 |
|  |  | mRNA 3'-end processing | 5.86E-04 |
|  |  | T cell receptor signaling pathway | 5.86E-04 |
|  |  | intracellular transport of virus | 5.86E-04 |
|  |  | tricarboxylic acid cycle | 8.61E-04 |
|  |  | peroxisome organization | 8.61E-04 |
|  |  | G2/M transition of mitotic cell cycle | 1.14E-03 |
|  |  | mismatch repair | 1.39E-03 |
|  |  | tRNA modification | 1.39E-03 |
|  |  | positive regulation of canonical Wnt signaling pathway | 1.65E-03 |
|  |  | RNA export from nucleus | 1.91E-03 |
|  |  | chromosome segregation | 2.16E-03 |
|  |  | nucleic acid phosphodiester bond hydrolysis | 2.41E-03 |
|  |  | positive regulation of telomere maintenance via telomerase | 2.65E-03 |
|  |  | mitochondrion morphogenesis | 2.89E-03 |
|  |  | protein N-linked glycosylation via asparagine | 3.90E-03 |
|  |  | double-strand break repair via homologous recombination | 4.34E-03 |
|  |  | positive regulation of gene expression, epigenetic | 4.34E-03 |
|  |  | centrosome cycle | 4.51E-03 |
|  |  | interstrand cross-link repair | 4.51E-03 |
|  |  | nucleotide-excision repair, DNA gap filling | 4.71E-03 |
|  |  | protein N-linked glycosylation | 5.12E-03 |
|  |  | telomere maintenance via semi-conservative replication | 5.12E-03 |
|  |  | regulation of translation | 5.56E-03 |
|  |  | RNA splicing, via transesterification reactions | 5.70E-03 |
|  |  | protein methylation | 5.70E-03 |
|  |  | cellular respiration | 6.59E-03 |
|  |  | CENP-A containing nucleosome assembly | 7.00E-03 |
|  |  | DNA replication initiation | 7.17E-03 |
|  |  | regulation of mitochondrial membrane potential | 7.51E-03 |
|  |  | RNA phosphodiester bond hydrolysis, endonucleolytic | 7.51E-03 |
|  |  | mRNA processing | 8.13E-03 |
|  |  | RNA secondary structure unwinding | 8.88E-03 |
|  |  | hydrogen ion transmembrane transport | 8.88E-03 |
|  |  | base-excision repair | 9.47E-03 |
|  |  | protein peptidyl-prolyl isomerization | 9.62E-03 |
|  |  | cell redox homeostasis | 9.98E-03 |
|  |  | positive regulation of viral transcription | 1.01E-02 |
|  |  | spindle organization | 1.25E-02 |
|  |  | protein heterotetramerization | 1.25E-02 |
|  |  | nucleotide-excision repair | 1.26E-02 |
|  |  | gluconeogenesis | 1.33E-02 |
|  |  | error-free translesion synthesis | 1.33E-02 |
|  |  | mitotic spindle assembly checkpoint | 1.50E-02 |
|  |  | protein oligomerization | 1.55E-02 |
|  |  | positive regulation of G1/S transition of mitotic cell cycle | 1.98E-02 |
|  |  | ciliary basal body-plasma membrane docking | 1.99E-02 |
|  |  | reactive oxygen species metabolic process | 2.06E-02 |
|  |  | telomere maintenance | 2.10E-02 |
|  |  | tumor necrosis factor-mediated signaling pathway | 2.30E-02 |
|  |  | Rac protein signal transduction | 2.38E-02 |

***Post-acute***

| **Post-acute (Up)** | | **Post-acute (Down)** | |
| --- | --- | --- | --- |
| viral transcription | 2.00E-05 | mitochondrial translational termination | 2.00E-05 |

***Late post-acute***

| **Late post-acute (Up)** | | **Late post-acute (Down)** | |
| --- | --- | --- | --- |
| nuclear-transcribed mRNA catabolic process, nonsense-mediated decay | 2.00E-05 | negative regulation of endopeptidase activity | 2.00E-05 |
| translation | 2.00E-05 | regulation of ion transmembrane transport | 1.38E-02 |
| translational initiation | 2.00E-05 | potassium ion transport | 1.84E-02 |
| SRP-dependent cotranslational protein targeting to membrane | 2.00E-05 |  |  |
| viral transcription | 2.00E-05 |  |  |
| T cell costimulation | 2.00E-05 |  |  |
| T cell activation | 2.00E-05 |  |  |
| positive regulation of I-kappaB kinase/NF-kappaB signaling | 2.00E-05 |  |  |
| regulation of immune response | 2.00E-05 |  |  |
| T cell receptor signaling pathway | 2.00E-05 |  |  |
| interferon-gamma-mediated signaling pathway | 2.00E-05 |  |  |
| type I interferon signaling pathway | 2.00E-05 |  |  |
| maturation of SSU-rRNA from tricistronic rRNA transcript (SSU-rRNA, 5.8S rRNA, LSU-rRNA) | 1.72E-03 |  |  |
| T cell differentiation | 1.72E-03 |  |  |
| peptidyl-tyrosine autophosphorylation | 1.72E-03 |  |  |
| transmembrane receptor protein tyrosine kinase signaling pathway | 2.75E-03 |  |  |
| regulation of tumor necrosis factor-mediated signaling pathway | 2.75E-03 |  |  |
| positive regulation of interleukin-4 production | 2.75E-03 |  |  |
| positive regulation of T cell differentiation | 2.75E-03 |  |  |
| cytokine production | 5.25E-03 |  |  |
| positive regulation of T cell proliferation | 7.18E-03 |  |  |
| protein autophosphorylation | 7.18E-03 |  |  |
| DNA recombination | 8.03E-03 |  |  |
| negative regulation of type I interferon production | 1.32E-02 |  |  |
| cellular response to amino acid starvation | 1.38E-02 |  |  |
| cellular response to lipopolysaccharide | 1.43E-02 |  |  |
| response to virus | 2.34E-02 |  |  |
| lipopolysaccharide-mediated signaling pathway | 2.34E-02 |  |  |
| positive regulation of calcium-mediated signaling | 2.34E-02 |  |  |
| B cell receptor signaling pathway | 2.34E-02 |  |  |
| cellular response to hydrogen peroxide | 2.34E-02 |  |  |
| regulation of signal transduction by p53 class mediator | 2.34E-02 |  |  |
| extrinsic apoptotic signaling pathway | 2.36E-02 |  |  |
| beta-catenin-TCF complex assembly | 2.36E-02 |  |  |
| B cell activation | 2.45E-02 |  |  |
| ribosomal small subunit biogenesis | 2.46E-02 |  |  |

## Table S5: List of significant gene sets in the acute-phase of the propensity-score matched sub-set of male and female patients.

| **Acute (Up)** | | **Acute (Down)** | |
| --- | --- | --- | --- |
| transmembrane receptor protein tyrosine kinase signaling pathway | 2.00E-05 | nuclear-transcribed mRNA catabolic process, nonsense-mediated decay | 2.00E-05 |
| axon guidance | 2.00E-05 | spliceosomal complex assembly | 2.00E-05 |
| peptidyl-serine phosphorylation | 2.00E-05 | spliceosomal snRNP assembly | 2.00E-05 |
| peptidyl-tyrosine phosphorylation | 2.00E-05 | maturation of SSU-rRNA from tricistronic rRNA transcript (SSU-rRNA, 5.8S rRNA, LSU-rRNA) | 2.00E-05 |
| protein autophosphorylation | 2.00E-05 | cytoplasmic translation | 2.00E-05 |
| interferon-gamma-mediated signaling pathway | 7.87E-03 | antigen processing and presentation of exogenous peptide antigen via MHC class I, TAP-dependent | 2.00E-05 |
| type I interferon signaling pathway | 7.87E-03 | mitochondrial electron transport, NADH to ubiquinone | 2.00E-05 |
| positive regulation of synapse assembly | 1.03E-02 | transcription-coupled nucleotide-excision repair | 2.00E-05 |
| animal organ morphogenesis | 1.50E-02 | nucleotide-excision repair, DNA incision, 5'-to lesion | 2.00E-05 |
| neutrophil chemotaxis | 1.50E-02 | termination of RNA polymerase II transcription | 2.00E-05 |
| negative regulation of inflammatory response | 1.50E-02 | mRNA export from nucleus | 2.00E-05 |
| homophilic cell adhesion via plasma membrane adhesion molecules | 2.39E-02 | translation | 2.00E-05 |
| regulation of phosphatidylinositol 3-kinase signaling | 2.39E-02 | translational initiation | 2.00E-05 |
| regulation of cell adhesion | 2.39E-02 | tRNA aminoacylation for protein translation | 2.00E-05 |
| odontogenesis of dentin-containing tooth | 2.39E-02 | protein folding | 2.00E-05 |
| cell fate commitment | 2.39E-02 | 'de novo' protein folding | 2.00E-05 |
| positive regulation of JNK cascade | 2.39E-02 | regulation of cellular amino acid metabolic process | 2.00E-05 |
| decidualization | 2.39E-02 | SRP-dependent cotranslational protein targeting to membrane | 2.00E-05 |
| homeostasis of number of cells | 2.39E-02 | protein targeting to mitochondrion | 2.00E-05 |
| positive regulation of ERK1 and ERK2 cascade | 2.39E-02 | ATP biosynthetic process | 2.00E-05 |
| cellular response to lipopolysaccharide | 2.39E-02 | sister chromatid cohesion | 2.00E-05 |
| cellular response to tumor necrosis factor | 2.39E-02 | RNA splicing | 2.00E-05 |
|  |  | negative regulation of G2/M transition of mitotic cell cycle | 2.00E-05 |
|  |  | viral transcription | 2.00E-05 |
|  |  | anaphase-promoting complex-dependent catabolic process | 2.00E-05 |
|  |  | SCF-dependent proteasomal ubiquitin-dependent protein catabolic process | 2.00E-05 |
|  |  | mitochondrial translation | 2.00E-05 |
|  |  | mitochondrial respiratory chain complex I assembly | 2.00E-05 |
|  |  | mitochondrial respiratory chain complex IV assembly | 2.00E-05 |
|  |  | nucleotide-excision repair, DNA incision | 2.00E-05 |
|  |  | NIK/NF-kappaB signaling | 2.00E-05 |
|  |  | ribosomal large subunit biogenesis | 2.00E-05 |
|  |  | cristae formation | 2.00E-05 |
|  |  | mitochondrial ATP synthesis coupled proton transport | 2.00E-05 |
|  |  | regulation of mRNA stability | 2.00E-05 |
|  |  | exonucleolytic nuclear-transcribed mRNA catabolic process involved in deadenylation-dependent decay | 2.00E-05 |
|  |  | nuclear import | 2.00E-05 |
|  |  | negative regulation of ubiquitin-protein ligase activity involved in mitotic cell cycle | 2.00E-05 |
|  |  | positive regulation of ubiquitin-protein ligase activity involved in regulation of mitotic cell cycle transition | 2.00E-05 |
|  |  | Wnt signaling pathway, planar cell polarity pathway | 2.00E-05 |
|  |  | chaperone-mediated protein folding | 2.00E-05 |
|  |  | regulation of transcription from RNA polymerase II promoter in response to hypoxia | 2.00E-05 |
|  |  | mitochondrial translational elongation | 2.00E-05 |
|  |  | mitochondrial translational termination | 2.00E-05 |
|  |  | global genome nucleotide-excision repair | 2.00E-05 |
|  |  | regulation of hematopoietic stem cell differentiation | 2.00E-05 |
|  |  | nucleotide-excision repair, DNA damage recognition | 5.30E-04 |
|  |  | fatty acid beta-oxidation | 5.30E-04 |
|  |  | mitochondrion organization | 5.30E-04 |
|  |  | tRNA processing | 5.30E-04 |
|  |  | ribosomal small subunit biogenesis | 5.30E-04 |
|  |  | regulation of gene silencing by miRNA | 5.30E-04 |
|  |  | transcription from RNA polymerase III promoter | 1.00E-03 |
|  |  | aerobic respiration | 1.00E-03 |
|  |  | maturation of SSU-rRNA | 1.00E-03 |
|  |  | ribosomal large subunit assembly | 1.38E-03 |
|  |  | nucleotide-excision repair, preincision complex stabilization | 1.38E-03 |
|  |  | nucleotide-excision repair, DNA incision, 3'-to lesion | 1.38E-03 |
|  |  | transcription elongation from RNA polymerase I promoter | 1.38E-03 |
|  |  | termination of RNA polymerase I transcription | 1.38E-03 |
|  |  | mitochondrial electron transport, cytochrome c to oxygen | 1.78E-03 |
|  |  | DNA damage response, detection of DNA damage | 1.78E-03 |
|  |  | transcription initiation from RNA polymerase I promoter | 2.12E-03 |
|  |  | RNA catabolic process | 2.12E-03 |
|  |  | heme biosynthetic process | 2.12E-03 |
|  |  | mitotic cell cycle | 2.43E-03 |
|  |  | tRNA export from nucleus | 2.43E-03 |
|  |  | mitochondrion morphogenesis | 2.43E-03 |
|  |  | error-prone translesion synthesis | 3.19E-03 |
|  |  | nucleobase-containing small molecule interconversion | 3.49E-03 |
|  |  | snRNA transcription from RNA polymerase II promoter | 3.49E-03 |
|  |  | regulation of glycolytic process | 4.59E-03 |
|  |  | nucleotide-excision repair, DNA duplex unwinding | 4.84E-03 |
|  |  | peroxisome organization | 4.84E-03 |
|  |  | mRNA 3'-end processing | 5.14E-03 |
|  |  | protein heterotetramerization | 5.44E-03 |
|  |  | mitotic spindle assembly checkpoint | 5.65E-03 |
|  |  | protein sumoylation | 5.65E-03 |
|  |  | mitotic nuclear envelope disassembly | 6.27E-03 |
|  |  | Fc-epsilon receptor signaling pathway | 6.89E-03 |
|  |  | tRNA modification | 8.16E-03 |
|  |  | nucleotide-excision repair, preincision complex assembly | 8.40E-03 |
|  |  | mitochondrial calcium ion transmembrane transport | 8.63E-03 |
|  |  | tricarboxylic acid cycle | 1.05E-02 |
|  |  | RNA metabolic process | 1.10E-02 |
|  |  | regulation of translational initiation | 1.18E-02 |
|  |  | branched-chain amino acid catabolic process | 1.30E-02 |
|  |  | regulation of cellular response to heat | 1.35E-02 |
|  |  | 7-methylguanosine mRNA capping | 1.36E-02 |
|  |  | chromosome segregation | 1.41E-02 |
|  |  | intracellular transport of virus | 1.42E-02 |
|  |  | positive regulation of canonical Wnt signaling pathway | 1.47E-02 |
|  |  | cellular respiration | 1.63E-02 |
|  |  | regulation of ubiquitin-protein ligase activity involved in mitotic cell cycle | 1.67E-02 |
|  |  | centrosome cycle | 1.68E-02 |
|  |  | DNA recombination | 1.72E-02 |
|  |  | RNA export from nucleus | 2.02E-02 |
|  |  | nucleic acid phosphodiester bond hydrolysis | 2.02E-02 |
|  |  | regulation of mitochondrial membrane potential | 2.20E-02 |
|  |  | protein N-linked glycosylation via asparagine | 2.20E-02 |
|  |  | regulation of G2/M transition of mitotic cell cycle | 2.48E-02 |
